# AbLWR: a context-aware listwise ranking framework for antibody-antigen binding affinity prediction via positive-unlabeled learning

**DOI:** 10.1093/bioinformatics/btag793

**Published:** 2026-10-23

**Authors:** Fan Xu, Zhi-An Huang, Haohuai He, Yidong Song, Wei Liu, Dongxu Zhang, Yao Hu, Jibin Wu, Kay Chen Tan

**Affiliations:** Department of Data Science and Artificial Intelligence, The Hong Kong Polytechnic University, Hong Kong SAR, China; Department of Computer Science, City University of Hong Kong (Dongguan), Dongguan, Guangdong 523808, China; Department of Computer Science, City University of Hong Kong, Hong Kong SAR, China; Department of Data Science and Artificial Intelligence, The Hong Kong Polytechnic University, Hong Kong SAR, China; School of Statistics and Data Science, Nankai University, Tianjin 300071, China; School of Public Health, Xiamen University, Xiamen, Fujian 361102, China; School of Public Health, Xiamen University, Xiamen, Fujian 361102, China; Department of Data Science and Artificial Intelligence, The Hong Kong Polytechnic University, Hong Kong SAR, China; Department of Data Science and Artificial Intelligence, The Hong Kong Polytechnic University, Hong Kong SAR, China; Department of Data Science and Artificial Intelligence, The Hong Kong Polytechnic University, Hong Kong SAR, China

## Abstract

**Motivation:**

Accurate prediction of antibody-antigen binding affinity is fundamental to therapeutic design and the rapid response to evolving pathogens. However, current computational approaches predominantly formulate this as a point-wise regression task. This paradigm struggles to discriminate subtle affinity differences among highly homologous variants and lacks the global context necessary to effectively prioritize top-tier candidates. Furthermore, the field is severely constrained by label sparsity. Although sequence data is abundant, experimentally verified affinities are scarce, leaving a massive pool of unverified antibody-antigen structural interactions unexploited by standard supervised paradigms.

**Results:**

We propose AbLWR (Antibody-antigen binding affinity Listwise Ranking), a novel framework that reformulates the conventional affinity regression task as a listwise ranking problem to directly optimize candidate prioritization. To mitigate label sparsity, AbLWR incorporates a Positive-Unlabeled (PU) learning mechanism, leveraging a dual-level contrastive objective and meta-optimized label refinement to distill robust structural representations from vast unlabeled data. To accommodate antigenic variation, we employ a homologous antigen sampling strategy coupled with a Multi-Head Self-Attention (MHSA) module, which explicitly models inter-sample dependencies within training lists to capture subtle affinity nuances. Extensive evaluations demonstrate that AbLWR significantly outperforms state-of-the-art sequence-, structure-, and complex-based baselines. It improves the Full Rank Accuracy (FRA) by approximately 8% compared to the strongest baseline in randomized cross-validation, and exhibits strong robustness in out-of-distribution scenarios involving distinct antigen and antibody clusters. Case studies on Influenza Viruses and Human Interleukin-33 further validate its practical utility. Specifically, AbLWR successfully tracks affinity drops caused by antigenic drift and immediately captures optimal binders in simulated wet-lab screening.

**Availability:**

Source code and datasets are freely available at https://github.com/Josie-xufan/AbLWR.

## 1 Introduction

Antibodies are central to the adaptive immune response, neutralizing pathogens via specific antigen binding mediated by complementarity-determining regions (CDRs) (Jin *et al*. 2022, Kim *et al*. 2023). The potency and efficacy of these interactions are typically evaluated using quantitative metrics, such as the dissociation constant ($K_{d}$), half-maximal inhibitory or effective concentrations (IC50/EC50), and escape fractions. Currently, the rapid evolution of antigens poses a significant challenge to antibody effectiveness (Tseng *et al*. 2020, Hie *et al*. 2021, Yang *et al*. 2023, Sun *et al*. 2025), creating a continually shifting antigenic landscape where emerging viral strains can evade the neutralizing effects of therapeutic antibodies (Garcia-Beltran *et al*. 2021, Zhang *et al*. 2021). Thus, developing methods to accurately identify and prioritize the most potent antibodies for specific, evolving antigens is of paramount importance.

Conventionally, binding affinity metrics are quantified through wet-lab experiments, such as Surface Plasmon Resonance (SPR), Enzyme-Linked Immunosorbent Assays (ELISA), and pseudovirus neutralization assays (Wang *et al*. 2022). However, these procedures are resource-intensive and time-consuming, particularly when antigenic drift necessitates multiple repeated experiments. Consequently, significant computational efforts have been directed toward binding affinity prediction (Frey *et al*. 2025). Historically, computational prediction relied heavily on physics-based simulations. Tools like Rosetta (Chaudhury *et al*. 2010) and FoldX (Schymkowitz *et al*. 2005) employ empirical force fields to estimate Gibbs free energy changes (ΔΔG) from the bound complex. While interpretable, these methods are computationally intensive and sensitive to structural noise.

Recently, the expansion of structural and interaction datasets, such as SAbDab (Dunbar *et al*. 2014) and SKEMPI (Jankauskaitė *et al*. 2019), has accelerated a paradigm shift toward data-driven approaches. Existing deep learning methodologies can be broadly classified into three paradigms based on their input modalities: sequence-, structure-, and complex-based methods. (1) *Sequence-based methods*. These approaches model affinity directly from amino acid sequences. Early efforts utilized 1D convolutional neural networks (CNNs) or recurrent neural networks (RNNs) to extract local motifs (Liberis *et al*. 2018, Mason *et al*. 2021, Huang *et al*. 2022). Recently, Protein Language Models (PLMs) have become dominant: general-purpose models like ESM-1b (Rives *et al*. 2021) and antibody-specific ones like AntiBERTy (Ruffolo *et al*. 2021) and MINT (Ullanat *et al*. 2026) leverage Masked Language Modeling to generate embeddings encapsulating evolutionary semantics. These representations are widely used for regression tasks (Gao *et al*. 2023). However, these methods inherently lack explicit spatial context, limiting their efficacy in capturing long-range interactions and discontinuous epitopes. (2) *Structure-based methods*. To address the limitations of sequence-only models, structure-based methods incorporate geometric data to enhance accuracy, typically deriving information from separate antibody or antigen structures. These approaches often employ geometric deep learning architectures to effectively capture local spatial features, such as 3D-CNNs (Wang *et al*. 2020) and graph neural networks (GNNs) (Bandara *et al*. 2025, Chen *et al*. 2025). Furthermore, the integration of advanced structure predictors like AlphaFold-3 (Abramson *et al*. 2024), IgFold (Ruffolo *et al*. 2023), and Boltz-1 (Wohlwend *et al*. 2025) enables these models to utilize high-quality predicted structures. (3) *Complex-based methods*. Deep learning complex-based methods operate directly on the 3D conformation of the bound state to capture interaction details. Recently, emerging methods like ANTIPASTI (Michalewicz *et al*. 2024) have been proposed to interpret complex stability. Despite their potential precision, these data-driven methods depend heavily on high-quality co-crystal structures or docked poses, which are often scarce and difficult to obtain for novel variants.

While these data-driven paradigms have significantly advanced the field, their overall predictive performance remains limited by two critical bottlenecks. First, existing models, which predominantly rely on point-wise regression, struggle to discriminate affinity levels among highly homologous Antibody-Antigen (Ab-Ag) pairs (Mason *et al*. 2021). As illustrated in Fig. 1a and c, multiple antibodies can bind to the identical antigen yet exhibit distinct affinity profiles. Treating these samples in isolation often fails to capture such subtle discrepancies in their relative binding strength. While pairwise ranking approaches (Liu *et al*. 2025) attempt to mitigate this, they are restricted to local comparisons, lacking the global context required to accurately prioritize top candidates. Second, the field suffers from severe label sparsity (Jiao *et al*. 2025). While sequence data is immense, experimentally verified affinity labels are scarce (Fig. 1b) (Dunbar *et al*. 2014, Jankauskaitė *et al*. 2019). Crucially, current fully supervised approaches largely overlook the vast potential of unlabeled Ab-Ag pairs, leaving a rich source of structural information unexploited.

**Figure 1:**
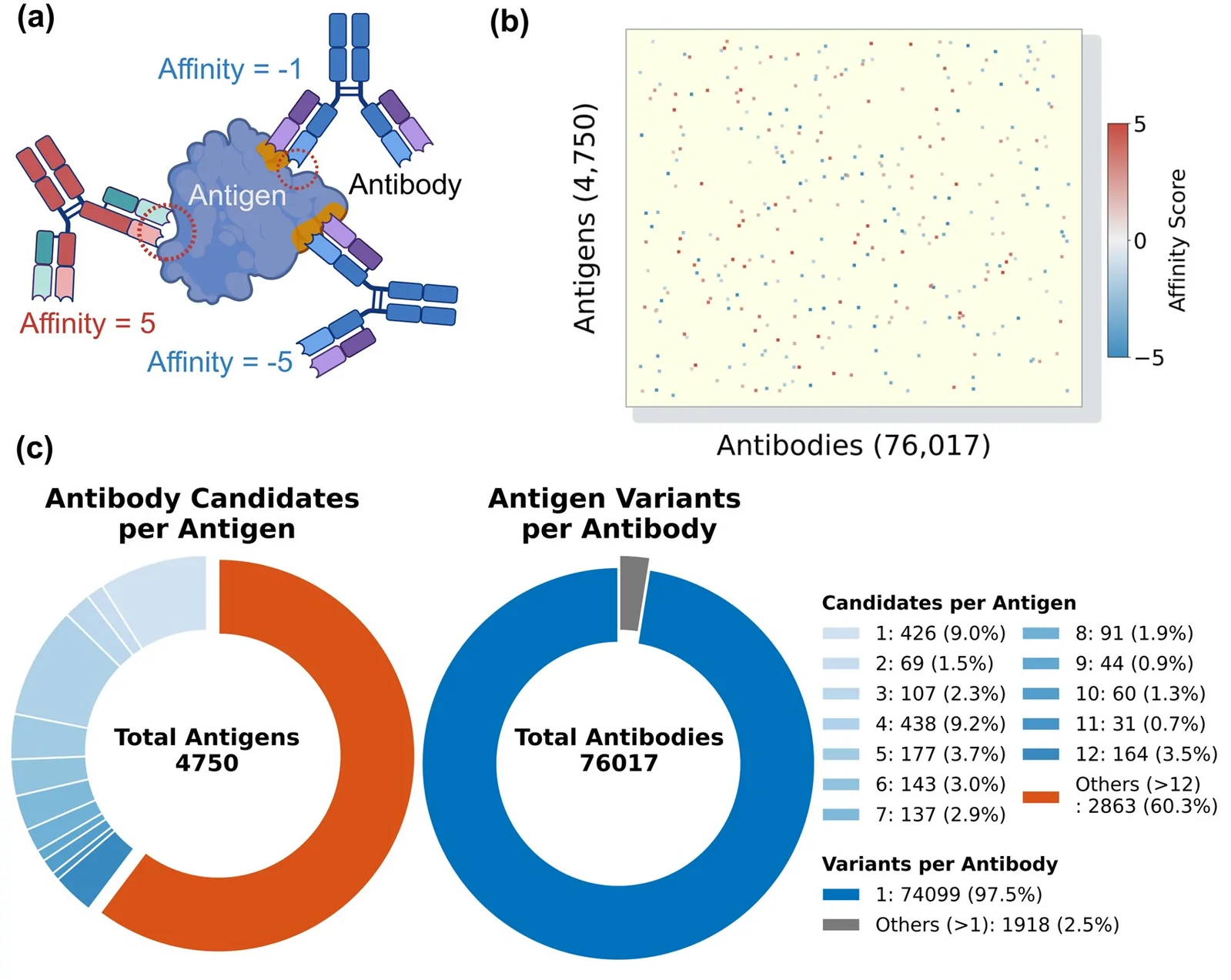
Key challenges in Ab-Ag binding affinity prediction. (a) Distinct affinity profiles of multiple antibodies binding to the same antigen. (b) The large reservoir of unlabeled data remains largely unexploited by existing supervised paradigms, compared with the immense volume of antibody and antigen sequences. (c) Analysis of existing binding affinity data from public datasets (refer to Supplementary Section S1.1, available as supplementary data at *Bioinformatics* online for details) reveals that most antigens are associated with different antibody candidates, whereas individual antibodies typically bind to a single specific antigen. Panel (a) includes elements created using BioRender.com.

Thus, we propose AbLWR, a novel two-stage framework that reformulates binding affinity prediction as a listwise ranking task. Rather than regressing absolute values, AbLWR aims to learn the relative order of Ab-Ag pairs based on binding strength. In the initial pre-training stage, we leverage Positive-Unlabeled (PU) learning to harness the abundance of unlabeled data for learning intrinsic structural representations. By treating experimentally verified pairs as positives and unverified pairs as unlabeled data, this module trains a classifier to stratify binding affinities into coarse-grained levels. In the subsequent ranking stage, we construct training lists comprising Ab-Ag pairs associated with identical or homologous antigens. To capture subtle affinity nuances, we develop a context-aware ranking module incorporating Multi-Head Self-Attention (MHSA) mechanism, which explicitly models the inter-dependencies among candidate pairs. Our contributions are summarized as follows:

- We propose AbLWR, the first attempt to reformulate affinity prediction from point-wise regression to listwise ranking. This shift aligns the optimization objective with candidate prioritization, capturing the relative affinity manifold more effectively than regression baselines.
- We introduce a semi-supervised PU learning strategy to tackle label sparsity. By distilling interaction priors from unlabeled Ab-Ag pairs, this approach yields robust representations that generalize well beyond limited experimental data.
- We devise a context-aware architecture incorporating MHSA module to model the global dependencies among candidate pairs. This mechanism enables the model to perform contrastive reasoning, directly distinguishing subtle residue variations across homologous sequences.
- Extensive evaluations on independent testing sets and task-specific splits demonstrate that AbLWR achieves state-of-the-art (SOTA) performance. Our results highlight the framework’s superior ability to identify high-affinity variants in lead optimization scenarios.

## 2 Methods and materials

This section outlines the methodology of AbLWR (see in Fig. 2). We begin by establishing the mathematical notations and problem definition in Section 2.1. Subsequently, Section 2.2 details the model architecture. Finally, training details including the learning objectives and the datasets are formulated in Section 2.3.

**Figure 2:**
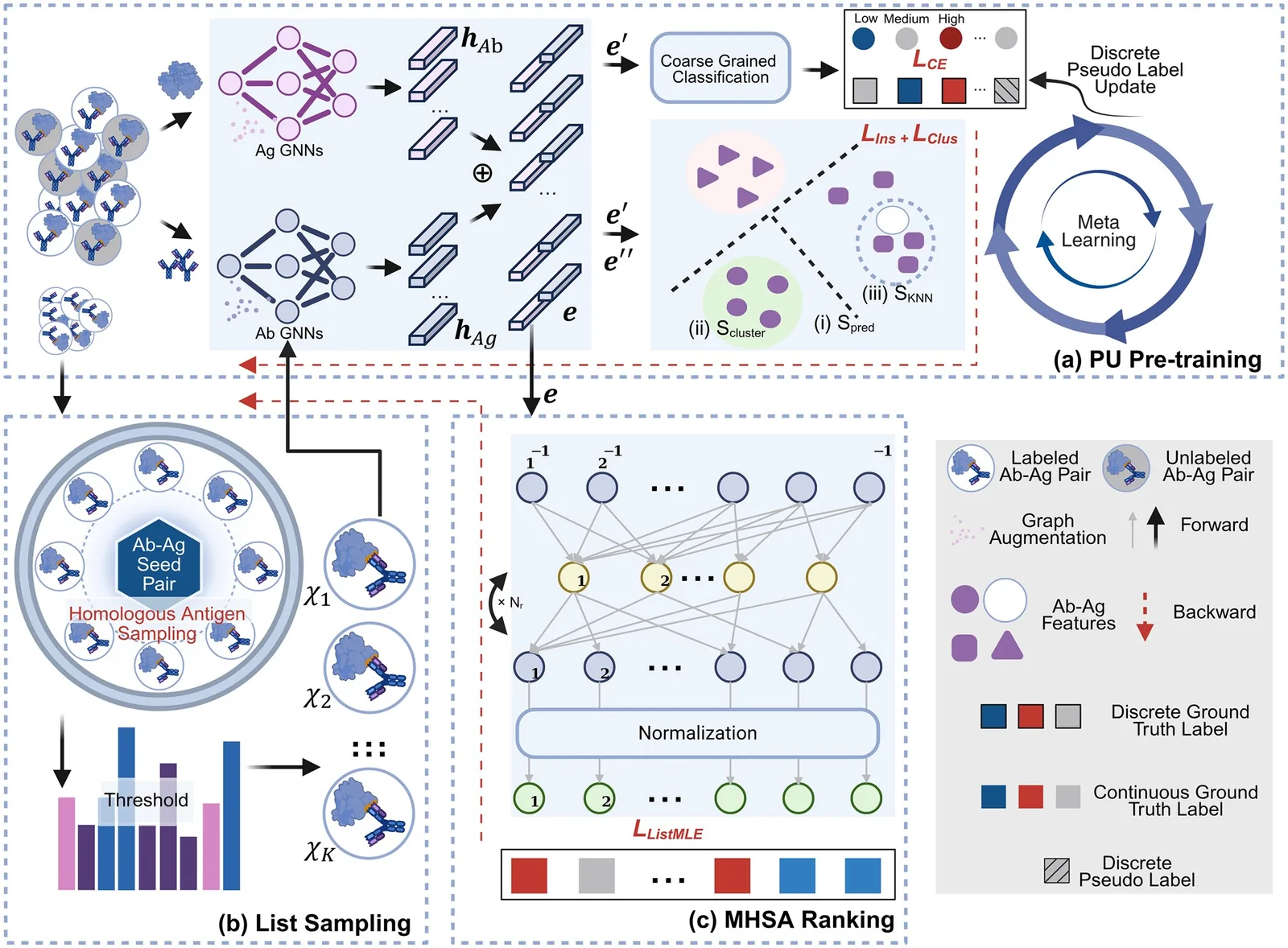
The overall framework of AbLWR. The architecture comprises three key components: (a) PU Pre-training: Dual GNN encoders learn robust representations from the combined dataset (D) by jointly optimizing a composite contrastive objective ($L_{\text{Ins}}+L_{\text{Clus}}$) and a cross entropy loss ($L_{\text{CE}}$) with meta-reweighted pseudo-labels. (b) List Sampling: A homologous sampling strategy constructs informative training lists T based on antigen sequence similarity ($\delta_{\text{seq}}$) and affinity margins ($y_{\text{cutoff}}$). (c) MHSA Ranking: The sampled lists are processed by MHSA mechanism to capture intra-list interactions via self-attention, yielding the final affinity ranking scores r. This figure was partly created with BioRender.com.

### 2.1 Problem definition

For a given antigen, we represent its primary sequence as $S_{\text{Ag}}=(b_{1},\ldots ,b_{L_{\text{Ag}}})$, where $L_{\text{Ag}}$ denotes the sequence length. For the antibody, we focus exclusively on the binding interface, representing it by its six CDRs with length $L_{\text{Ab}}$: $S_{\text{Ab}}=(a_{1},\ldots ,a_{L_{\text{Ab}}})$. To incorporate geometric constraints, we leverage pre-trained structure prediction models: IgFold (Ruffolo *et al*. 2023) for antibodies and ESMFold (Lin *et al*. 2023) for antigens. The resulting 3D structures are represented as point clouds $X_{\text{Ab}}=\{x_{1},\ldots ,x_{L_{\text{Ab}}}\}$ and $X_{\text{Ag}}=\{x_{1},\ldots ,x_{L_{\text{Ag}}}\}$, where $x_{j}\in R^{C\times 3}$ denotes the coordinates of *C* atoms in the *j*-th residue. Formally, we define a single Ab-Ag pair instance as the tuple: $X≜(S_{\text{Ab}},S_{\text{Ag}},X_{\text{Ab}},X_{\text{Ag}})$.

We frame the binding affinity prediction problem as a listwise ranking task. Let $T=\{X_{1},\ldots ,X_{K}\}$ denote a query list comprising *K* Ab-Ag pairs (where K=5), and $y={\left[y_{1},\ldots ,y_{K}\right]}^{⊤}$ be the corresponding ground-truth binding affinities. Our goal is to learn a scoring function $f:T\to R^{K}$ that predicts ranking scores $r={\left[r_{1},\ldots ,r_{K}\right]}^{⊤}$ such that the ranking order of r is consistent with y.

### 2.2 Model architecture

#### 2.2.1 Ab-Ag GNN encoders

We further model the Ab-Ag pair X as residue-level molecular graphs, $G_{\text{Ab}}=(N_{\text{Ab}},E_{\text{Ab}})$ and $G_{\text{Ag}}=(N_{\text{Ag}},E_{\text{Ag}})$. Edges (u,v)∈E are established based on the coordinates X if the Euclidean distance between any non-hydrogen atoms in residues *u* and *v* is less than 4.5Å (Pittala and Bailey-Kellogg 2020). Initial node features $H^{\left(0\right)}$ are derived by encoding the sequences S with PLMs: IgFold for antibodies and ESM-2 for antigens.

To independently model the distinct structural topologies of the antibody and antigen, we employ dual Graph Convolutional Network (GCN) encoders, $\phi_{\text{Ab}}$ and $\phi_{\text{Ag}}$ (Kipf 2016). Both encoders utilize a two-layer architecture to project heterogeneous features into a shared latent space $R^{d_{\text{out}}}$ (where $d_{\text{out}}=64$), yielding refined node representations $H_{\text{Ab}}$ and $H_{\text{Ag}}$. These residue-level features are aggregated into global graph representations, $h_{\text{Ab}}$ and $h_{\text{Ag}}$, via global mean pooling. The final embedding for the pair X is formed by concatenating these vectors: $e=[h_{\text{Ab}}||h_{\text{Ag}}]\in R^{2d_{\text{out}}}$.

#### 2.2.2 PU pre-training

To enhance representation learning, we employ a PU pre-training strategy (Fig. 2a) on the combined dataset $D=D_{L}\cup D_{U}$, where $D_{L}$ and $D_{U}$ denote the labeled and unlabeled Ab-Ag pairs, respectively. As a prerequisite, we also generate two augmented views via stochastic graph perturbations: a weakly augmented representation $e^{\prime }$ and a strongly augmented representation $e^{\prime \prime }$ (see Supplementary Section S3.3, available as supplementary data at *Bioinformatics* online for details). Given the continuous affinity y∈R, we first discretize it into a categorical label $y^{\text{cls}}\in \{-1,0,1\}$, representing high, medium, and low affinity levels, respectively. To facilitate a unified training objective, we define a target variable ${\tilde{y}}^{\text{cls}}$ for each Ab-Ag pair. For labeled data ($X\in D_{L}$), we set ${\tilde{y}}^{\text{cls}}=y^{\text{cls}}$, using the ground truth; for unlabeled data ($X\in D_{U}$), ${\tilde{y}}^{\text{cls}}$ is the pseudo-label initialized as 0 and refined by the model’s prediction.

We optimize a multi-task objective on the combined dataset, integrating supervised classification with self-supervised contrastive learning. The classification objective utilizes the weakly augmented view $e_{i}^{\prime }$ and a classifier $f_{\text{cls}}$ to predict the affinity class probabilities ${\hat{y}}_{i}^{\mathrm{cls}}=f_{\text{cls}}(e_{i}^{\prime })$ and the cross-entropy loss ($L_{\text{CE}}$) is used to minimize the discrepancy between predictions and the targets. Simultaneously, we minimize an instance-level contrastive loss to align the $\ell_{2}$-normalized weak and strong views:


(1) $$\begin{array}{c} L_{\text{Ins}}=-\frac{1}{B}\sum_{i=1}^{B}\;\text{log}\;\frac{e^{\left(e_{i}^{\prime }\cdot e_{i}^{\prime \prime }/\tau \right)}}{\Omega_{i}},\\ \text{where}\;\Omega_{i}=e^{\left(e_{i}^{\prime }\cdot e_{i}^{\prime \prime }/\tau \right)}+\sum_{\overset{j\neq i}{\;}}\left[e^{\left(e_{i}^{\prime }\cdot e_{j}^{\prime \prime }/\tau \right)}+e^{\left(e_{i}^{\prime }\cdot e_{j}^{\prime }/\tau \right)}\right]. \end{array}$$

Following a warm-up period, we refine the representation space to group pairs with similar binding patterns by incorporating a cluster-aware loss $L_{\text{Clus}}$. For each query $e_{i}^{\prime }$, we construct a robust positive set $P_{i}$ by integrating the strategies of consensus verification and geometric preservation (Long *et al*. 2024). This loss encourages the query to align with its semantic neighbors:


(2) $$L_{\text{Clus}}=-\frac{1}{B}\sum_{i=1}^{B}\frac{1}{\left|P_{i}\right|}\sum_{p\in P_{i}}\;\text{log}\;\frac{e^{e_{i}^{\prime }\cdot p/\tau }}{\Omega_{i}}.$$

Subsequently, we adopt a meta-learning strategy (Ren *et al*. 2018) to refine the initial pseudo-labels assigned to $D_{U}$. Let $B_{L}$ and $B_{U}$ denote the indices of labeled and unlabeled data, respectively. We construct the unified target matrix ${\tilde{Y}}_{\text{cls}}\in R^{B\times N_{c}}$ (where $N_{c}=3$) by stacking the current targets ${\left\{{\tilde{y}}_{i}^{\text{cls}}\right\}}_{i=1}^{B}$. We then introduce a learnable perturbation matrix $\Delta \in R^{B\times N_{c}}$ to generate soft targets via ${\tilde{Y}}_{\text{cls}}+\Delta$. The optimization proceeds in a bi-level manner. We first perform a virtual gradient step on the model parameters using the soft targets, and then seek the optimal perturbation $\Delta^{*}$ that minimizes the loss on a clean validation batch $D_{L}^{\text{val}}$. Guided by this optimization, we derive refined discrete pseudo-labels ${\tilde{Y}}_{\text{cls}}^{\text{meta}}$. Finally, to stabilize training, we update the targets using an exponential moving average (EMA) with the momentum coefficient of β:


(3) $${\tilde{Y}}_{\text{cls}}^{\left(t+1\right)}=\beta \;{\tilde{Y}}_{\text{cls}}^{\left(t\right)}+(1-\beta ){\tilde{Y}}_{\text{cls}}^{\text{meta}}.$$

The detailed derivation of the positive set construction and the bi-level optimization are available in Supplementary Section S4, available as supplementary data at *Bioinformatics* online.

#### 2.2.3 List sampling

Following pre-training, we fine-tune the model using a listwise ranking objective. To capture subtle affinity differences, we construct informative ranking lists $T=\{X_{1},\ldots ,X_{K}\}$ using a homologous antigen sampling strategy (illustrated in Fig. 2b). Formally, for a randomly sampled seed pair $X_{\text{se}}$ with antigen sequence $S_{\text{Ag}}^{\text{se}}$ and ground-truth affinity $y_{\text{se}}$, we retrieve a candidate pool $P_{\text{se}}$ from labeled dataset $D_{L}$. The pool is restricted to candidates that exhibit high antigen homology with the seed yet significant affinity divergence:


(4) $$\begin{array}{c} P_{\text{se}}=\{X_{i}\in D_{L}\;|\text{Sim}(S_{\text{Ag}}^{i},S_{\text{Ag}}^{\text{se}})\geq \delta_{\text{seq}}\\ \wedge \left|y_{\text{se}}-y_{i}\right|>y_{\text{cutoff}}\}, \end{array}$$

where Sim(·) denotes sequence similarity, $\delta_{\text{seq}}$ is the homology threshold, and $y_{\text{cutoff}}$ enforces a minimum margin to mitigate measurement noise. Finally, K−1 pairs are selected from $P_{\text{se}}$ and combined with $X_{\text{se}}$ to form the list T. Specific sampling configurations for different data splits and auxiliary strategies for global robustness are also detailed in Supplementary Section S2, available as supplementary data at *Bioinformatics* online.

#### 2.2.4 MHSA ranking

To map the structural embeddings to scalar affinity ranking scores, we employ a ranking module based on MHSA mechanism. Specifically, we adopt the Induced Set Attention Block (ISAB) variant (Lee *et al*. 2019) to mediate the information flow, by introducing *M* learnable inducing points as a reference frame. Formally, we construct the input matrix $E_{T}\in R^{K\times 2d_{\text{out}}}$ by stacking the embeddings of the *K* pairs in the list T. These embeddings are then processed through $N_{r}$ layers of ISAB (see Supplementary Section S4, available as supplementary data at *Bioinformatics* online for mathematical details). Finally, the context-aware features $Z^{\left(L\right)}\in R^{K\times d_{r}}$ are projected to scalar scores via a linear layer: $r=Z^{\left(L\right)}w+b$, where $w\in R^{d_{r}}$ and b∈R are learnable parameters, and $r\in R^{K}$ represents the ranking scores for the *K* pairs.

### 2.3 Training

#### 2.3.1 Dataset and split

We curated Ab-Ag pairs from eight public repositories, derived from the compilation by Liu *et al*. (2025) (details in Supplementary Section S2, available as supplementary data at *Bioinformatics* online). Each entry comprises paired variable heavy and light chains alongside their corresponding antigen sequences. Following the established protocol, binding affinity measurements were standardized to the equilibrium dissociation constant ($K_{d}$) and log-transformed ($\text{log}\;K_{d}$) to address measurement heterogeneity. Subsequently, the dataset was subsampled to 60 620 pairs for training and evaluation. We employed three splitting strategies to rigorously assess model generalization: (1) standard Random Split with 5-fold cross-validation; (2) Ag-based Split to test performance across antigen types; and (3) Ab-based Split using CDR clustering to assess robustness against antibody variations. Detailed splitting protocols are provided in Supplementary Section S2.2, available as supplementary data at *Bioinformatics* online. Additionally, our case studies utilize the influenza virus dataset from Momont *et al.* (Momont *et al*. 2023) and the human interleukin-33 (IL-33) dataset from Wang *et al.* (Wang *et al*. 2025) (see Supplementary Section S9, available as supplementary data at *Bioinformatics* online).

#### 2.3.2 Training objectives

We adopt a two-stage optimization strategy. The pre-training phase minimizes a composite objective $L_{\text{pre}}=L_{\text{CE}}+L_{\text{Ins}}+L_{\text{Clus}}$ to learn robust representations. In the fine-tuning stage, we optimize the ranking performance using the ListMLE loss, which maximizes the likelihood of the correct permutation based on the Plackett-Luce model (Xia *et al*. 2008). Since smaller values indicate stronger binding, the target permutation $\pi =[\pi_{1},\ldots ,\pi_{K}]$ is obtained by sorting the labels in ascending order: $y_{\pi_{1}}\leq y_{\pi_{2}}\leq \cdots \leq y_{\pi_{K}}$. The objective is defined as:


(5) $$L_{\text{ListMLE}}=-\sum_{i=1}^{K}\;\text{log}\;\left(\frac{\;\text{exp}(-r_{\pi_{i}})}{\sum_{j=i}^{K}\;\text{exp}\;(-r_{\pi_{j}})}\right).$$

## 3 Experiments and results

In this section, we present a comprehensive evaluation of AbLWR across diverse data splitting strategies. We begin with the standard Random Split in Section 3.1, followed by more challenging scenarios involving distinct clusters in Section 3.2. Furthermore, we conduct ablation studies to verify the contribution of AbLWR’s key components in Section 3.3. Finally, we demonstrate the practical applicability of AbLWR in real-world biological settings through case studies in Section 3.4.

### 3.1 Comparison with SOTA methods under Random Split

#### 3.1.1 Baseline models

We benchmark AbLWR against a comprehensive suite of SOTA methods. We categorize these baselines into: (1) sequence-based: MINT (Ullanat *et al*. 2026) and AntiBERTy (Ruffolo *et al*. 2021); (2) structure-based: GraphDTA (Nguyen *et al*. 2021) and AbRank (Liu *et al*. 2025); (3) complex-based: ANTIPASTI (Michalewicz *et al*. 2024) and PBEE (Chaves *et al*. 2025); and (4) traditional methods, including the physics-based FoldX (Schymkowitz *et al*. 2005) and the tree-based LambdaMART (Burges 2010). Detailed training configurations are provided in Supplementary Section S6.1, available as supplementary data at *Bioinformatics* online.

#### 3.1.2 Evaluation metrics

We employ five metrics to assess ranking performance: (1) Full Rank Accuracy (FRA), reflecting overall ranking quality; (2) Kendall’s τ (Kτ), measuring the ordinal association between predicted and ground-truth values; (3) Pairwise Rank Accuracy (PRA) and (4) Pairwise AUC (PAU), quantifying the percentage of correctly ordered sample pairs; and (5) Precision@1 (P@1), measuring the accuracy of the highest-ranked prediction.

#### 3.1.3 Experimental results

Table 1 summarizes the quantitative comparison results on the Random Split setting. Our proposed method, AbLWR, consistently outperforms all competing baselines across all five evaluation metrics. Specifically, in terms of the challenging FRA metric, AbLWR achieves 20.74%, establishing a significant lead over the second-best performing method, GraphDTA (12.75%). Similarly, for the ranking correlation metric Kτ, our model attains 0.59, clearly surpassing the strongest baseline (0.54). It is worth noting that AbLWR also demonstrates superior stability, as evidenced by the relatively low standard deviations. Regarding the computationally intensive methods evaluated on the subsampled dataset, our corresponding model AbLWR* maintains its dominance with an FRA of 17.14%, outperforming both complex-based and traditional baselines.

**Table 1:** Performance comparison on the Random Split.

Category	Model	FRA (%)	Kτ	PRA (%)	PAU (%)	P@1 (%)
Sequence-based	MINT	1.11 ± 0.09	0.07 ± 0.00	53.33 ± 0.16	53.33 ± 0.16	23.12 ± 0.62
	AntiBERTy	8.00 ± 0.43	0.43 ± 0.02	71.38 ± 0.80	71.38 ± 0.80	44.11 ± 2.18
Structure-based	AbRank	5.63 ± 2.78	0.31 ± 0.11	65.40 ± 5.61	65.40 ± 5.61	39.64 ± 5.65
	GraphDTA	12.75 ± 0.70	0.54 ± 0.01	76.87 ± 0.36	76.87 ± 0.36	53.38 ± 0.87
Complex-based	ANTIPASTI*	0.00^a^ ± 0.00	−0.07 ± 0.07	46.24 ± 3.69	46.24 ± 3.69	17.96 ± 4.42
	PBEE*	0.00^a^ ± 0.00	−0.03 ± 0.03	40.56 ± 3.20	48.52 ± 2.72	17.86 ± 7.88
Traditional	LambdaMART	6.36 ± 0.24	0.38 ± 0.01	68.18 ± 0.64	68.79 ± 0.63	39.04 ± 0.48
	FoldX*	2.04 ± 1.44	0.01 ± 0.09	50.24 ± 4.36	50.27 ± 4.35	17.14 ± 2.74
**Ours**	**AbLWR**	**20.74** ± **0.45**	**0.59** ± **0.01**	**79.31** ± **0.52**	**79.49** ± **0.51**	**57.26** ± **0.56**
	**AbLWR** *	**17.14** ± **5.88**	**0.57** ± **0.05**	**78.16** ± **2.54**	**78.30** ± **2.65**	**53.47** ± **5.28**

We employ 5-fold cross-validation (mean ± std) for all methods. Due to computational costs, the methods of FoldX, ANTIPASTI, and PBEE (marked with*) were evaluated on a subsampled dataset.

a Near-zero FRA reflects sequence-to-complex error accumulation.

Accordingly, AbLWR* reports our model’s performance on this specific subset to ensure a fair comparison. All metrics are in percentage (%) except for Kτ, and higher values indicate better performance. Best results are highlighted in **bold**.

### 3.2 Generalization on distinct antigen and antibody clusters

Table 2 extends our evaluation to the Ag-based and Ab-based splits, designed to assess model robustness under severe distribution shifts. For consistency, we employ the same baseline methods and evaluation metrics as in Section 3.1. In the Ag-based scenario, AbLWR demonstrates strong performance with an FRA of 10.28%, representing a significant lead over baseline methods. This advantage is also evident in other ranking metrics, confirming the model’s robustness on distinct antigen distributions.

**Table 2:** Performance comparison on Ag-based and Ab-based Splits.

Category	Model	FRA (%)	Kτ	PRA (%)	PAU (%)	P@1 (%)
**Ag-based Split**						
Sequence-based	MINT	0.67	0.00	49.63	49.63	21.93
	AntiBERTy	6.95	0.46	72.69	72.69	46.16
Structure-based	AbRank	1.61	0.15	57.20	57.20	24.21
	GraphDTA	9.76	0.50	74.94	74.94	48.53
Complex-based	ANTIPASTI^∗^	0.00^a^	−0.01	49.39	49.39	22.45
	PBEE^∗^	0.00^a^	0.01	40.61	52.24	22.45
Traditional	LambdaMART	2.56	0.22	60.67	60.73	28.32
	FoldX^∗^	2.04	0.10	54.49	54.49	36.73
**Ours**	**AbLWR**	**10.28**	**0.51**	**75.25**	**75.62**	**50.10**
	**AbLWR** ^∗^	**12.24**	**0.54**	**76.53**	**77.03**	**59.18**
**Ab-based Split (Cluster 5)**						
Sequence-based	MINT	0.82	−0.02	49.03	49.03	19.30
	AntiBERTy	0.89	−0.01	46.11	46.11	15.77
Structure-based	AbRank	4.15	0.35	67.68	67.68	41.43
	GraphDTA	0.72	−0.01	49.55	49.55	18.01
Complex-based	ANTIPASTI^∗^	0.00	0.04	51.84	51.84	16.33
	PBEE^∗^	0.00^a^	0.12	50.82	53.27	18.37
Traditional	LambdaMART	0.99	0.07	44.99	43.22	30.10
	FoldX^∗^	0.00^a^	0.02	50.82	50.82	20.41
**Ours**	**AbLWR**	**5.66**	**0.37**	**68.60**	**68.62**	**46.35**
	**AbLWR** ^∗^	**4.08**	**0.38**	**68.98**	**68.98**	**44.90**
**Ab-based Split (Cluster 1)**						
Sequence-based	MINT	0.74	−0.03	48.56	48.56	18.86
	AntiBERTy	1.64	0.11	55.32	55.32	27.72
Structure-based	AbRank	2.53	0.25	62.33	62.33	38.23
	GraphDTA	2.92	0.24	62.01	62.01	36.46
Complex-based	ANTIPASTI^∗^	0.00^a^	0.08	54.08	54.08	24.49
	PBEE^∗^	0.02^a^	0.02	44.29	52.38	14.29
Traditional	LambdaMART	1.25	0.09	53.37	54.48	23.59
	FoldX^∗^	2.04	−0.04	47.76	47.76	18.37
**Ours**	**AbLWR**	**3.58**	**0.25**	**62.66**	**62.68**	**39.21**
	**AbLWR** ^∗^	**8.16**	**0.28**	**64.08**	**64.08**	**38.78**

The Ag-based split corresponds to the single most divergent cluster, while the Ab-based split comprises the two most distinct clusters.

a Near-zero FRA reflects sequence-to-complex error accumulation.

∗ Models marked with an asterisk were evaluated on a subsampled dataset.

The Ab-based splits (Cluster 5 and Cluster 1) impose a stricter test, leading to a universal decline in performance metrics. Nevertheless, AbLWR retains its primacy in identifying the most potent binders. Specifically, our method maintains a clear advantage in FRA (5.66% and 3.58%) and P@1 (46.35% and 39.21 %). This suggests that AbLWR is precise in prioritizing the top candidates, a critical trait for practical antibody discovery. Finally, comparisons on the subsampled dataset reinforce this trend, with AbLWR* significantly exceeding FoldX, ANTIPASTI, and PBEE particularly in the challenging Ab-based clusters where traditional methods struggle.

### 3.3 Ablation studies

To validate the effectiveness of the proposed components in AbLWR, we conducted a comprehensive ablation study focusing on two key aspects: homologous antigen sampling and model architecture. As detailed in Table 3, we compare the full model against five variants: (1) *w/AbRankSampling*: replaces our sampling strategy with the one used in AbRank (Liu *et al*. 2025), which considers affinity divergence but neglects antigen homology; (2) *w/PU Init. (Non-binder)*: initializes the pseudo-label ${\tilde{y}}^{\text{cls}}$ as non-binder; (3) *w/o PU Init.*: removes the PU initialization and relies solely on random initialization; (4) *w/o Rank. ListMLE*: reformulates the objective as an absolute affinity regression task using Mean Squared Error (MSE) loss instead of the ranking-based ListMLE; (5) *w/o Rank. MHSA*: substitutes the MHSA mechanisms with simple Multilayer Perceptrons (MLPs). The results in Table 3 demonstrate that all proposed components contribute significantly to the final performance. Most notably, replacing the ranking loss with MSE regression (*w/o Rank. ListMLE*) causes the most significant performance degradation, with FRA falling from 21.28% to 3.29%. This underscores that modeling relative order is far more effective than absolute regression for this task. Similarly, removing PU initialization entirely (*w/o PU Init.*) leads to a sharp decline to 5.52%, highlighting the critical role of our initialization strategy in leveraging unlabeled data. Furthermore, initializing the pseudo-labels ${\tilde{y}}^{\text{cls}}$ as non-binders (*w/PU Init. (Non-binder)*) reduces the FRA to 18.72%. This demonstrates that treating unconfirmed samples as neutral is more appropriate than rigidly categorizing them as non-binders, as it better accommodates potential hidden positives within the unlabeled set. In terms of model architecture, substituting the MHSA mechanisms with MLPs (*w/o Rank. MHSA*) results in a notable drop to 13.90%, validating its capability to capture complex dependencies. Finally, our homologous antigen sampling strategy outperforms the baseline sampling (*w/AbRankSampling*) by a substantial margin (8.73% in FRA), confirming the necessity of incorporating biological homology into the training process. Two additional ablation studies to directly verify the effectiveness of ISAB and listwise design are reported in the Supplementary Section S8.

**Table 3:** Ablation study on the test set.

Model	FRA (%)	Kτ	PRA (%)	PAU (%)	P@1 (%)
**AbLWR (Ours)**	**21.28**	**0.60**	**79.96**	**80.14**	**57.73**
*Sampling Strategy*					
w/AbRankSampling	12.55	0.52	75.73	75.90	52.92
*Component Ablation*					
w/PU Init. (Non-binder)	18.72	0.55	77.52	77.70	53.31
w/o PU Init.	5.52	0.34	66.08	66.25	41.67
w/o Rank. ListMLE	3.29	0.56	59.17	59.30	31.55
w/o Rank. MHSA	13.90	0.25	77.65	77.83	55.70

We evaluate the impact of different components and strategies.

### 3.4 Case studies on influenza viruses and human interleukin-33

To evaluate the practical effectiveness of AbLWR, we conducted case studies on the influenza virus and human IL-33. For the influenza study, we curated 15 Ab-Ag pairs involving three antibodies and five antigens (detailed in Supplementary Section S9, available as supplementary data at *Bioinformatics* online) (Momont *et al*. 2023), which yielded 3003 unique K=5 lists through exhaustive combination. Regarding the human IL-33 study, we analyzed the I7 antibody alongside 19 candidates derived from affinity maturation (Wang *et al*. 2025), resulting in a total of 15 504 lists. The ranking performance (Kτ) is summarized in Table S11, available as supplementary data at *Bioinformatics* online and the detailed visualizations of high-correlation lists (Kendall’s τ>0.4) are provided in Figure S3, available as supplementary data at *Bioinformatics* online.

We further analyzed the influenza virus dataset from two complementary perspectives. First, we evaluated ranking consistency across 18 test lists derived from the homologous antigen sampling strategy. As shown in Fig. 3, the heatmap demonstrates strong alignment: high-affinity pairs (green) are consistently ranked at the top, while low-affinity ones (red) are relegated to the bottom. This observation is supported by the adjacent bar chart, where the majority of tuples exhibit high Kτ scores. Second, we examine FNI17 antibody, which exhibits potent binding to Influenza B (IVB) but significantly reduced affinity for mutated Influenza A (IVA) strains emerging after 2015 (Wan *et al*. 2019, Powell and Pekosz 2020). As shown in Fig. 4a and b, AbLWR effectively discriminates between these viral types. Despite subtle sequence variations, the model correctly predicts the affinity drop in mutated strains, confirming its sensitivity to structural changes driven by viral evolution.

**Figure 3:**
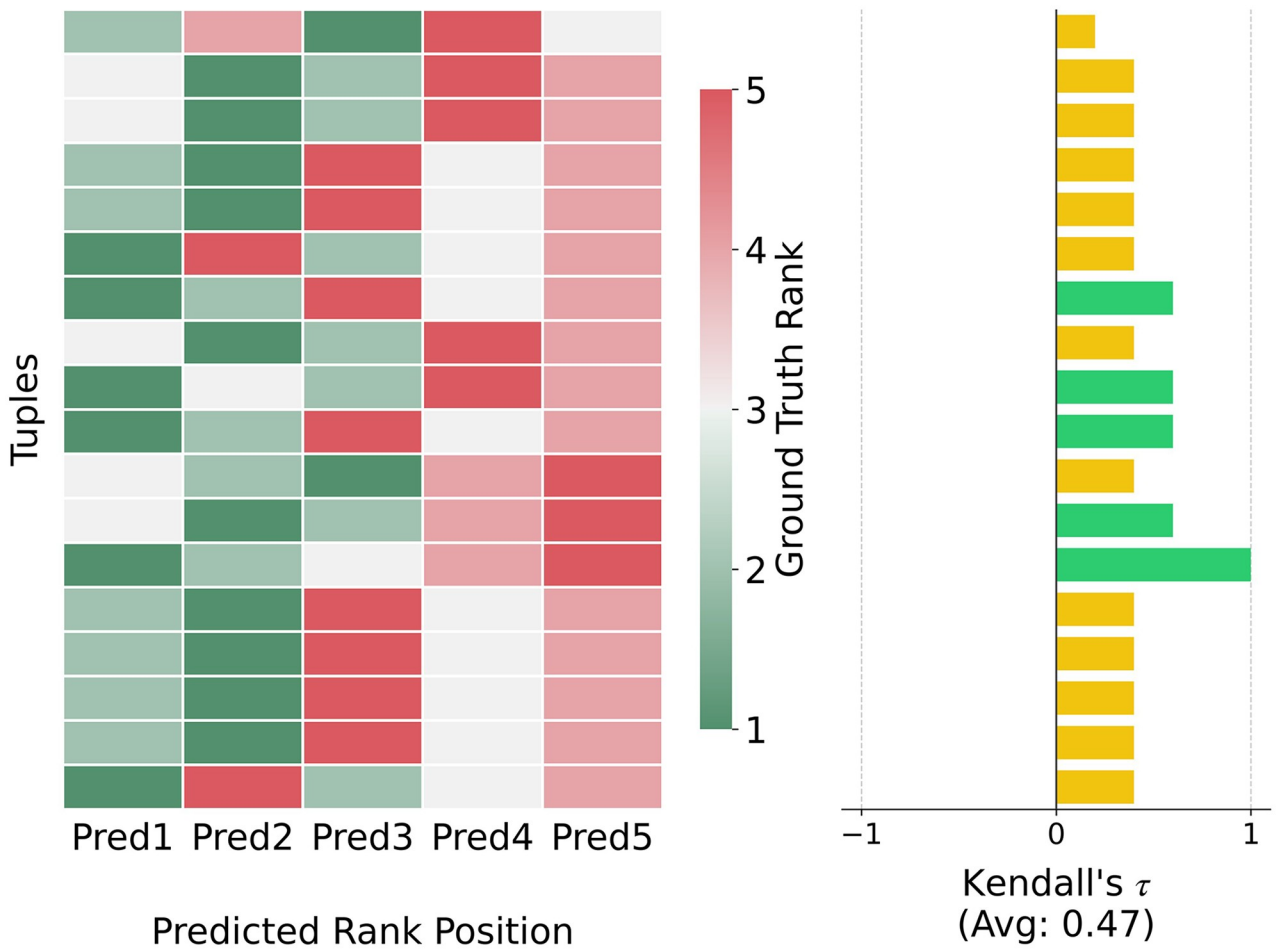
Visualization of ranking predictions on 18 sampled lists. The heatmap displays the ground truth ranks mapped to the model’s predicted positions. Each row represents a test list colored by their ground truth rank, and columns denote the predicted order. The bar chart shows the corresponding Kτ calculated for each list row.

**Figure 4:**
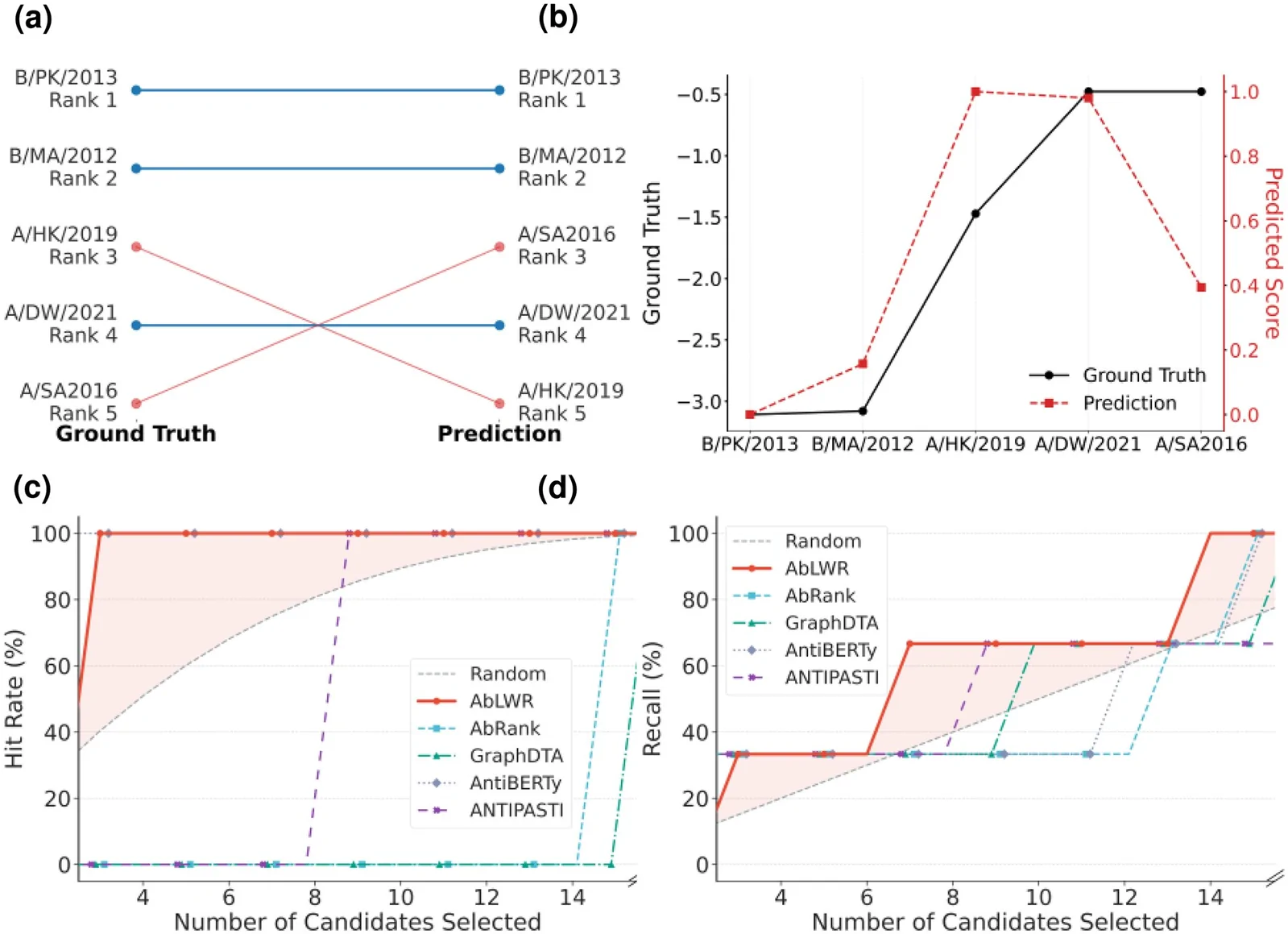
Case studies on FNI17 antibody mechanism and candidates screening for IL-33 antigen. (a) Comparison of ground truth ranks and predicted ranks. Lines connect the same strain across the two rankings. (b) Dual-axis plot showing the ground truth values and predicted scores for the corresponding viral strains. (c) Hit rate curve for identifying the top-1 binder to the IL-33 antigen. (d) Recall curve showing the percentage of top-3 IL-33 binders retrieved against the number of candidates screened.

Using the human IL-33 dataset, we demonstrate AbLWR’s efficiency in accelerating wet-lab screening. While identifying the optimal binder typically requires exhaustive testing of all 20 candidates, AbLWR significantly streamlines this process. In comparison to the representative data driven baselines, which struggle to surpass random selection in early rounds (Fig. 4c), AbLWR secures a hit immediately upon the initial selection. Furthermore, it efficiently captures all three best binders within 14 screening rounds (Fig. 4d). These results demonstrate that AbLWR substantially reduces experimental burden while maintaining high discovery success.

## 4 Conclusion

We proposed AbLWR, a listwise framework that redefines binding affinity prediction as a ranking task. By integrating PU learning to mitigate label sparsity and a context-aware architecture to model inter-sample dependencies, AbLWR effectively captures relative binding strengths. Empirical results confirm its superiority over existing SOTA methods and the potential in experimental laboratory settings, such as wet-lab screening. Despite these promising results, the current list sampling strategy is decoupled from the training pipeline and the list size *K* is a static hyperparameter. Future work will integrate the sampling process directly into the learning loop and develop adaptive mechanisms to dynamically determine *K* based on data complexity.

## Supplementary Material

btag793_Supplementary_Data

## Data Availability

The processed dataset comprising 60 620 antibody-antigen pairs, along with the source code used in this study, are freely available at the GitHub repository: https://github.com/Josie-xufan/AbLWR. The raw data were originally collected from eight publicly available databases, as detailed in the Supplementary Information, available as supplementary data at *Bioinformatics* online.
