# Supplementary material for: AbLWR: a context-aware listwise ranking framework for antibody-antigen binding affinity prediction via positive-unlabeled learning
Source: Bioinformatics. 2026 Oct 23;42(11):btag793. doi: 10.1093/bioinformatics/btag793 (PMC13728850; doi:10.1093/bioinformatics/btag793)
Supplement: Supplementary file 1 [file btag793_supplementary_data.pdf]

Supplementary Information

October 20, 2026

S1 Symbols and Notations

Table S1: Summary of main symbols and notations.

**Symbol**	**Description**
Input representation	
$\mathbf{S}_{\mathrm{Ag}}, \mathbf{S}_{\mathrm{Ab}}$	Antigen and antibody sequences; antibody is represented by six CDRs.
$L_{\mathrm{Ag}}, L_{\mathrm{Ab}}$	Sequence lengths of antigen and antibody CDRs.
$\mathbf{X}_{\mathrm{Ag}}, \mathbf{X}_{\mathrm{Ab}}$	3D structural coordinates of antigen and antibody CDRs.
$\mathcal{X}$	A single antibody-antigen pair.
Graph representation	
$\mathcal{G}_{\mathrm{Ag}}, \mathcal{G}_{\mathrm{Ab}}$	Residue-level graphs of antigen and antibody.
$\mathcal{N}, \mathcal{E}$	Node and edge sets of a graph.
$\mathbf{A}, \tilde{\mathbf{A}}$	Adjacency matrix and normalized adjacency matrix.
$\phi_{\mathrm{Ag}}, \phi_{\mathrm{Ab}}$	GNN encoders for antigen and antibody.
$\mathbf{h}_{\mathrm{Ag}}, \mathbf{h}_{\mathrm{Ab}}$	Graph-level representations after pooling.
d_{out}	Output dimension of each GNN encoder.
$\mathbf{e}, \mathbf{e}', \mathbf{e}''$	Original, weakly augmented, and strongly augmented pair embeddings.
Datasets	
$\mathcal{D}, \mathcal{D}_L, \mathcal{D}_U$	Combined, labeled, and unlabeled datasets used in PU pretraining.
$\mathcal{D}_{\mathrm{train}}, \mathcal{D}_{\mathrm{val}}, \mathcal{D}_{\mathrm{test}}$	Training, validation, and test partitions.
$\mathcal{B}, \mathcal{B}_L, \mathcal{B}_U$	Mini-batch, labeled samples, and unlabeled samples.
PU pretraining	
$y, y^{\mathrm{cls}}, \tilde{y}^{\mathrm{cls}}$	Continuous affinity, discretized class label, and pseudo-label.
$\tilde{\mathbf{Y}}_{\mathrm{cls}}, \tilde{\mathbf{Y}}_{\mathrm{cls}}^{\mathrm{meta}}$	Target matrix and meta-refined pseudo-label matrix.
$f_{\mathrm{cls}}, \hat{\mathbf{y}}^{\mathrm{cls}}$	PU classifier and predicted class probabilities.
$\boldsymbol{\Delta}, \boldsymbol{\Delta}^*$	Label perturbation and optimized perturbation for meta-refinement.
α, β, τ	Meta-learning rate, EMA momentum, and contrastive temperature.
$\mathcal{P}_i$	Positive set for sample i in contrastive learning.
$\mathcal{L}_{\mathrm{CE}}, \mathcal{L}_{\mathrm{Ins}}, \mathcal{L}_{\mathrm{Clus}}$	Classification, instance-level contrastive, and cluster-aware losses.
$\mathcal{L}_{\mathrm{pre}}$	Overall PU pretraining objective.
Listwise ranking	
$\mathcal{T}, K$	Ranking list and list size.
$\mathbf{y}, \mathbf{r}$	Ground-truth affinity vector and predicted score vector.
r_i	Predicted score of the i-th pair.
π, π_i	Ground-truth permutation and its i-th ranked index.
$\mathcal{X}_{\mathrm{se}}, \mathcal{P}_{\mathrm{se}}$	Seed pair and candidate pool for homologous sampling.
$\mathrm{Sim}(\cdot), \delta_{\mathrm{seq}}, y_{\mathrm{cutoff}}$	Sequence similarity, homology threshold, and affinity margin threshold.
$\mathcal{L}_{\mathrm{ListMLE}}$	ListMLE ranking loss.
$\mathbf{E}_{\mathcal{T}}$	Pair-embedding matrix of a ranking list.
d_r, N_r, M	Ranking hidden dimension, number of ISAB layers, and inducing points.
N	Number of test query lists.

S2 Details for Data Collection and Analysis

S2.1 Data Collection

The distribution of Ab-Ag pairs across different data sources is available from Liu et al. (Liu et al., 2025). Brief descriptions are provided below:

- **RBD-escape (Greaney et al., 2022):** Derived from Deep Mutational Scanning (DMS) experiments, this dataset quantifies how mutations in the SARS-CoV-2 Receptor Binding Domain (RBD) affect antibody binding.
- **CATNAP (Yoon et al., 2015):** A database focused on HIV-1, compiling neutralizing antibody data against HIV-1 Envelope pseudoviruses.
- **AlphaSeq (Engelhart et al., 2022):** Contains quantitative interaction measurements between designed antibody libraries and specific antigens, generated using the high-throughput AlphaSeq platform.

- **AbCoV (Rawat et al., 2022):** A curated repository specifically for coronaviruses, aggregating binding data for antibodies and nanobodies against SARS-CoV-2, SARS-CoV-1, and MERS-CoV.
- **SKEMPI 2.0 (Jankauskaitė et al., 2019):** A database of thermodynamic binding data ($\Delta\Delta G$ and K_d) derived from literature for protein complexes with known 3D structures.
- **AbSci (Shanehsazzadeh et al., 2023):** Originates from wet-lab screening campaigns for de novo antibody design, providing binding scores for antibody variants against targets like HER2.
- **SAbDab (Dunbar et al., 2014):** The Structural Antibody Database. We utilized the subset of Ab-Ag complexes that have experimentally measured binding affinities annotated in the database.
- **OVA-binders (Goldstein et al., 2019):** Derived from a massively parallel screening study identifying antibody variants that bind to the model antigen Chicken Ovalbumin (OVA).

From the initial collection of 342,356 Ab-Ag pairs, we excluded 1,059 entries that lacked binding affinity labels. Furthermore, to address inconsistent annotations, we identified duplicate pairs sharing identical sequences but differing in affinity values. Those with a $\log K_d$ discrepancy exceeding 1.0 were discarded. This filtering process resulted in 340,296 retained pairs, involving 4,750 antigens and 76,017 antibodies as shown in fig. 1 (c). Specific statistics information of the selected samples is presented in fig. S1, demonstrating the significant heterogeneity in binding affinity among different antibodies targeting the same antigen. Subsequently, following the protocol established by Liu et al. (Liu et al., 2025), we subsampled the dataset to mitigate bias from dominant antigen groups and verified the validity of all sequences. The final dataset comprises 60,620 Ab-Ag pairs and has been deposited in Zenodo.

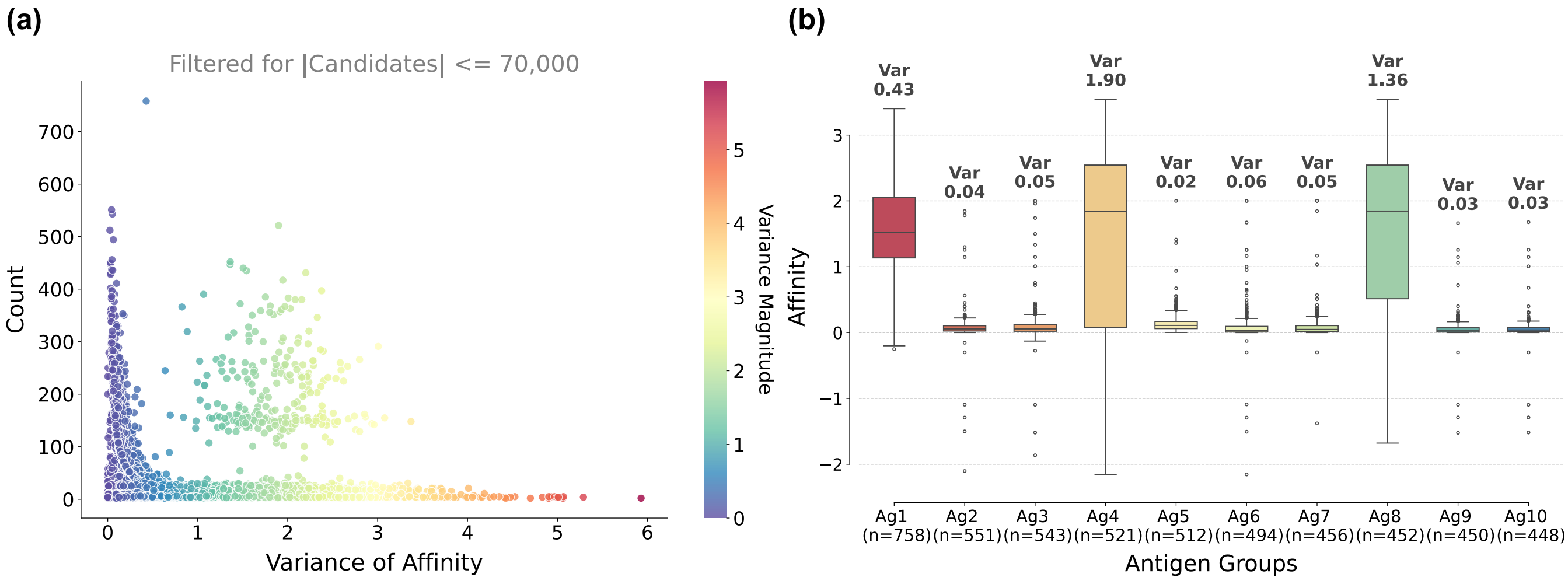

Figure S1: **Binding affinity statistics.** (a) Affinity variance per antigen. (b) Affinity distribution for the top 10 most represented antigen groups.

S2.2 Data Split

To comprehensively assess the model's performance across different scenarios, we designed three distinct data splitting strategies, covering random partitioning and biological sequence segmentation.

Random Split. Five-fold cross-validation was employed to assess the model's generalization on randomly split samples, where sequence similarity between training and test sets is not explicitly controlled.

Ag-based Split. To evaluate the model's capability of generalizing to novel antigen targets (OOD, Out-of-Distribution generalization), we performed clustering on all antigen sequences (fig. S2 (a)). In real-world antibody engineering, models are often required to predict binding affinities for antigens that differ significantly from the training distribution, such as emerging viral variants or novel protein families (Cox et al., 2023). To rigorously simulate this distribution shift, we identified the most divergent cluster (Cluster 0), which exhibits the largest spectral distance from the remaining dataset. We allocated 70% of samples from this cluster to the test set, merging the remaining 30% with other clusters for training. This partition constructs a few-shot OOD scenario, where the model has limited exposure to the target domain.

Ab-based Split. To assess the model's ability to prioritize high-affinity pairs among antibodies with distinct paratopes, we clustered antibody sequences based on their CDRs (fig. S2 (b)) and selected the two most divergent clusters (Cluster 5 and Cluster 1). Similar to the antigen split, 70% of these distinct antibodies were reserved for testing. This split evaluates whether the model can effectively screen for binders that possess low sequence identity to the majority of the training data, a critical requirement for discovering diverse therapeutic candidates.

To facilitate comparison with baseline methods that require explicit binding state prediction, which are computationally intensive, we employed a subsampling strategy. For each test partition, we randomly selected 50 listwise examples from the corresponding test set using a fixed random seed of 42. The sampling was performed at the list level without replacement and was not stratified. For the random-split setting, each fold has its own test partition. Therefore, the 50 lists were sampled independently within each fold, and different folds do not share a common set of 50 lists. The same deterministic sampling procedure and random seed were used for the Ab-based and Ag-based split settings. Within each fold or split setting, the saved list indices were shared by the baselines, ensuring that all the baseline methods were evaluated on exactly the same listwise samples. To make the subsampled evaluation reproducible, we released the source test-set files at `https://github.com/Josie-xufan/AbLWR` and `https://doi.org/10.5281/zenodo.20069587`. The statistical information of the subset is recorded in table S2.

Table S2: Statistics of the subsampled test sets used to evaluate baseline methods that require explicit binding state prediction.

**Split setting**	**Test lists**	**Selected lists**	**Complex occurrences**	**Unique complexes**
Random split, fold 1	13,030	50	250	245
Random split, fold 2	13,102	50	250	246
Random split, fold 3	13,014	50	250	248
Random split, fold 4	12,901	50	250	250
Random split, fold 5	13,095	50	250	248
Ab-based split, cluster 5	4,027	50	250	246
Ab-based split, cluster 0	4,074	50	250	244
Ag-based split	15,325	50	250	248

Note: Complex occurrences count all $50 \times K = 250$ positions in the selected listwise samples. A complex may appear in more than one selected list. Therefore, the number of unique complexes can be smaller than 250.

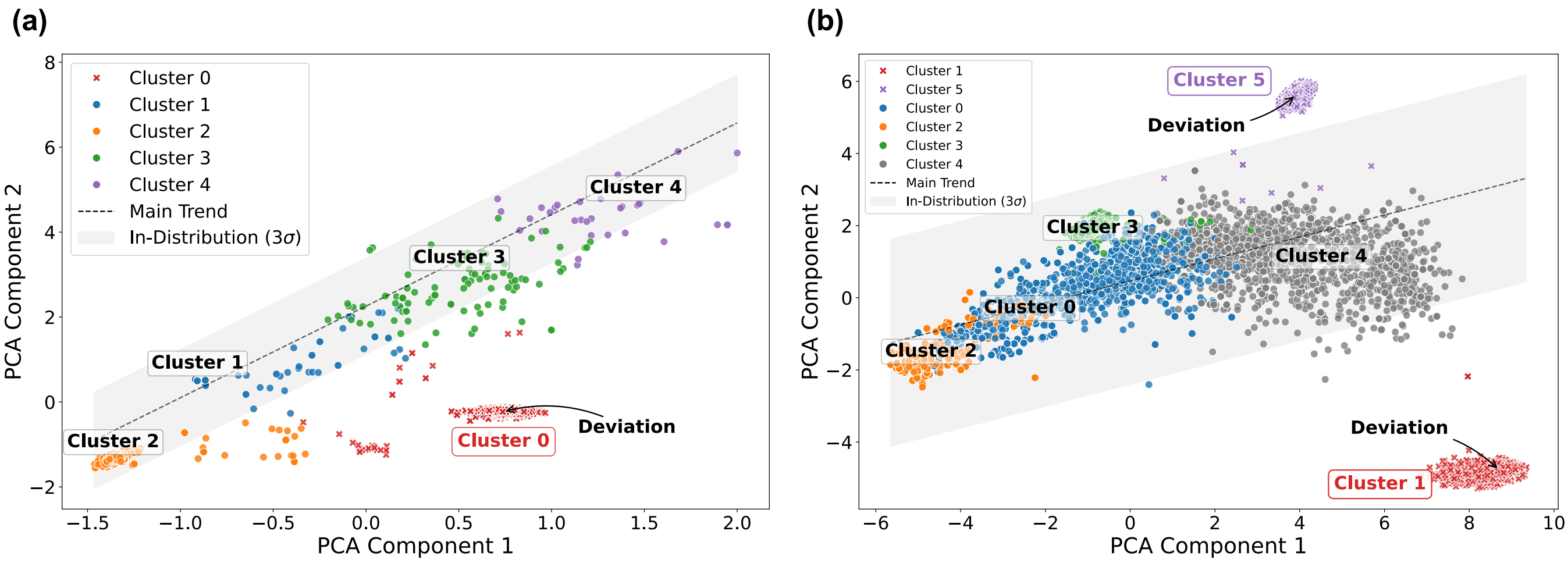

Figure S2: **Visualization of sequence clustering.** (a) Antigen sequence clustering. *Cluster 0* is identified as a divergent group distinct from the main distribution and is used to define the Ag-based split. (b) Antibody sequence clustering based on CDRs. *Cluster 1* and *Cluster 5* exhibit significant deviation from the main trend and are selected for the Ab-based split. In both scenarios, these distinct clusters serve as the primary source for the test sets to evaluate model generalization capabilities.

S2.3 Data Sampling

We tailor the sampling composition and hyperparameters to the specific objectives of each data split:

- **Random and Ag-based Splits:** In these scenarios, the model is required to generalize to diverse antigen structures. Relying solely on homologous sampling could cause the model to degenerate into a set of disjoint local rankers. Therefore, we construct the training data using a balanced 1:1 mixture of homologous lists and heterogeneous lists. These heterogeneous lists, formed by pairs with distinct antigen sequences, force the model to look beyond specific antigen scaffolds and learn universal physicochemical interaction patterns that apply across different biological families. Here, we set the affinity margin threshold $y_{\text{cutoff}} = 0.5$.
- **Ab-based Split:** For this split, we exclusively employ homologous antigen sampling and increase the threshold to $y_{\text{cutoff}} = 1.0$. This design aligns with the antibody lead selection scenario, where the goal is to identify the most potent binders from a diverse pool of candidates targeting the specific antigen (as seen in fig. 1 (c)). In the complex antibody space, small affinity differences often arise from noisy or non-generalizable sequence variations. By enforcing a larger margin between all pairs, we provide the model with high-confidence ranking signals, ensuring it learns robust relationships that can transfer to unseen antibody clusters.

S3 Details for Ab-Ag GNN Encoders

S3.1 Graph Construction and Feature Initialization

We independently model the antibody and antigen as graphs $\mathcal{G} = (\mathcal{N}, \mathcal{E})$, where nodes $\mathcal{N}$ represent amino acid residues. The edge set $\mathcal{E}$ is constructed based on spatial proximity derived from the 3D coordinates $\mathbf{X}$. Specifically, an edge (u, v) is established between residue u and residue v if the minimum Euclidean distance between any of their non-hydrogen atoms is less than a threshold of 4.5 Å(Pittala and Bailey-Kellogg, 2020). This is formally defined as:

$$(u, v) \in \mathcal{E} \iff \min_{\mathbf{c} \in \mathbf{x}_u, \mathbf{c}' \in \mathbf{x}_v} \|\mathbf{c} - \mathbf{c}'\|_2 < 4.5, \tag{S1}$$

where $\mathbf{x}_u$ and $\mathbf{x}_v$ denote the sets of atomic coordinates for residues u and v, respectively. To capture rich semantic information, we initialize node features $\mathbf{H}^{(0)}$ by encoding the primary sequences $\mathbf{S}$ using pre-trained PLMs. For the antibody, we utilize IgFold (Ruffolo et al., 2023) to encode the CDR sequences:

$$\mathbf{H}^{(0)}_{\text{Ab}} = \text{IgFold}(\mathbf{S}_{\text{Ab}}) \in \mathbb{R}^{L_{\text{Ab}} \times 512}. \tag{S2}$$

For the antigen, we employ ESM-2 (Lin et al., 2023) to encode the antigen sequence:

$$\mathbf{H}^{(0)}_{\text{Ag}} = \text{ESM-2}(\mathbf{S}_{\text{Ag}}) \in \mathbb{R}^{L_{\text{Ag}} \times 1280}. \tag{S3}$$

S3.2 GNN Encoders

The graph encoders are implemented using the PyTorch Geometric library. As detailed in eq. (S1), the adjacency matrix $\mathbf{A}$ is constructed based on a spatial distance threshold of 4.5 Å, where the distance between two residues is defined as the minimum Euclidean distance between any pair of their non-hydrogen atoms. We employ `GCNConv` layers with symmetric normalization. Let $\tilde{\mathbf{A}}$ denote the normalized adjacency matrix. The layer-wise propagation is defined as $\mathbf{H}^{(l+1)} = \sigma(\tilde{\mathbf{A}}\mathbf{H}^{(l)}\mathbf{W}^{(l)})$, where $\mathbf{W}^{(l)}$ is the learnable weight matrix and $\sigma(\cdot)$ is the ReLU activation function. The architecture comprises two parallel branches, the antibody encoder and the antigen encoder, both utilizing a two-layer GCN to project high-dimensional features into a shared 64-dimensional latent space.

S3.3 Graph Augmentation

To construct diverse views required for the PU learning framework, we employ weak and strong augmentations to introduce stochastic perturbations to both structure features and node features.

Weak Augmentation. The weak augmentation strategy focuses solely on minor structural perturbations to preserve the semantic integrity of the protein graph while introducing necessary variance. We apply random edge dropout, where edges are removed from the adjacency matrix with a low probability of 0.1.

Strong Augmentation. The strong augmentation strategy applies more aggressive perturbations to both structure and node features to prevent the model from overfitting to specific local patterns. Structural Perturbation: We apply random edge dropout with a higher probability of 0.3. Node Feature Masking: We randomly mask node features to force the model to rely on contextual information with the probability of 0.3.

S4 Details for PU Pre-training

S4.1 Construction of $\mathcal{D}_U$

We first collected the available antibody-antigen complexes with affinity annotations. The resulting labeled dataset was partitioned into training, validation, and test subsets according to the predefined random, antigen-based, or antibody-based split protocol. Let

$$\mathcal{D}_{\text{obs}} = \mathcal{D}_{\text{train}} \cup \mathcal{D}_{\text{val}} \cup \mathcal{D}_{\text{test}} \tag{S4}$$

denote the set of all observed labeled antibody-antigen pairs. The unlabeled pool $\mathcal{D}_U$ was constructed by randomly sampling antibody-antigen combinations from the available antibody and antigen candidate sets. During sampling, every pair in $\mathcal{D}_{\text{obs}}$ was excluded. The size of the unlabeled pool was set to the number of labeled training pairs: $|\mathcal{D}_U| = |\mathcal{D}_{\text{train}}|$. No affinity value was assigned to the sampled pairs in $\mathcal{D}_U$. They were used only as unlabeled examples in the pretraining procedure. Importantly, affinity labels from the validation and test subsets were never used to compute the pretraining loss.

S4.2 Discretization of Continuous Affinity Values

As mentioned in the main text, we categorize the continuous binding affinity values (y) into three coarse-grained classes (y^{cls}) during the pre-training phase. The thresholds for this discretization are set at ± 0.5. The selection of these thresholds was strictly data-driven, designed to reflect the empirical statistical distribution of the affinity values in our dataset while optimizing the model's discriminative learning. Specifically, our dataset comprises a total of 60,620 samples, with a median affinity of 0.40 and a standard deviation of 1.25. We summarize the exact data distribution across these three intervals in table S3.

Table S3: Distribution of discretized affinity classes.

**Affinity (y)**	**Label (y^{cls})**	**Biological Meaning**	**Proportion (%)**
$y < -0.5$	-1	High Affinity	25.96
$-0.5 \leq y \leq 0.5$	0	Medium Affinity	26.85
$y > 0.5$	1	Low Affinity	47.19

In Ab-Ag interactions, true high-affinity binders are inherently scarce. Setting the threshold at -0.5 successfully isolates approximately the top 26% of our dataset, ensuring a high-quality positive class. Furthermore, the median of our dataset (0.40) falls squarely within the $[-0.5, 0.5]$ interval. By designating this range as the "Medium" class, we accurately capture the statistical center of the data. Crucially, this coarse-grained discretization prevents noisy gradients from minor continuous variations, allowing the model to focus on definitive "High" and "Low" extremes.

S4.3 Positive set $\mathcal{P}_i$ Construction

To construct a robust positive set $\mathcal{P}_i$ that mitigates the noise from individual views, we integrate strategies of consensus verification and geometric preservation.

Consensus Verification. This strategy relies on two complementary candidate sets derived from cluster alignment ($\mathcal{S}_{\text{cluster}}$) and prediction consistency ($\mathcal{S}_{\text{pred}}$). First, we apply K-means clustering on the representations to assign a prototype ρ_i to each sample i and the cluster-based set is defined as $\mathcal{S}^{(i)}_{\text{cluster}} = \{j \mid \rho_j = \rho_i, j \neq i\}$. Second, to ensure task alignment, the prediction consistency set groups samples with identical predicted affinity classes: $\mathcal{S}^{(i)}_{\text{pred}} = \{j \mid \arg\max f_{\text{cls}}(\mathbf{e}'_j) = \arg\max f_{\text{cls}}(\mathbf{e}'_i), j \neq i\}$. Ultimately, only samples present in both sets are incorporated to ensure mutual verification.

Geometric Preservation To preserve local geometric structure, we select the k-nearest neighbors based on cosine similarity: $\mathcal{S}^{(i)}_{\text{KNN}} = \text{Top-}k(\{j \mid j \neq i\}, \mathbf{e}'_i \cdot \mathbf{e}'_j)$.

Finally, the robust positive set is derived by taking the union of the verified consensus and the geometric preservation: $\mathcal{P}_i = (\mathcal{S}_{\text{cluster}} \cap \mathcal{S}_{\text{pred}}) \cup \mathcal{S}_{\text{KNN}}$.

S4.4 Bi-Level Meta-Optimization

The core objective of the meta-optimization is to learn an optimal label perturbation $\boldsymbol{\Delta}$ for the unlabeled data by leveraging a small, clean validation set $\mathcal{D}^{\text{val}}_L$. To strictly preserve experimental ground truth, we enforce $\boldsymbol{\Delta}_i = \mathbf{0}$ for all $i \in \mathcal{B}_L$. Let $\theta = \{\phi_{\text{Ab}}, \phi_{\text{Ag}}, f_{\text{cls}}\}$ denote the joint parameters of the graph encoders and the classifier. First, we perform a virtual gradient step on the entire classification branch using the soft targets:

$$\theta'(\boldsymbol{\Delta}) = \theta - \alpha \nabla_\theta \mathcal{L}_{\text{CE}}(f_{\text{cls}}(\mathbf{e}'_\theta), \tilde{\mathbf{Y}}_{\text{cls}} + \boldsymbol{\Delta}), \tag{S5}$$

Algorithm 1 Bi-Level Meta-Optimization

Input: Data Batch $\mathcal{B} = \mathcal{B}_L \cup \mathcal{B}_U$, Validation Data $\mathcal{D}_L^{\text{val}}$, Model Parameters θ, Targets $\tilde{\mathbf{Y}}_{\text{cls}}^{(t)}$, Hyperparams α, β.
Virtual Update: Compute θ' with current soft targets:
  $\theta' \leftarrow \theta - \alpha \nabla_\theta \mathcal{L}_{\text{CE}}(f_{\text{cls}}(\mathbf{e}'_\theta), \tilde{\mathbf{Y}}_{\text{cls}}^{(t)} + \mathbf{\Delta})$
Meta-Optimization: Find $\mathbf{\Delta}^*$ minimizing validation loss:
  $\mathbf{\Delta}^* \leftarrow \arg\min_{\mathbf{\Delta}} \mathcal{L}_{\text{CE}}(f_{\text{cls}}(\mathbf{e}_{\theta'}), \tilde{\mathbf{Y}}_{\text{cls}}^{\text{val}})$
Update: Enforce $\mathbf{\Delta}_i^* = \mathbf{0}$ for $i \in \mathcal{B}_L$ and update targets:
  $\tilde{\mathbf{Y}}_{\text{cls}}^{\text{meta}} \leftarrow \text{OneHot}(\arg\max(\tilde{\mathbf{Y}}_{\text{cls}}^{(t)} + \mathbf{\Delta}^*))$
  $\tilde{\mathbf{Y}}_{\text{cls}}^{(t+1)} \leftarrow \beta \tilde{\mathbf{Y}}_{\text{cls}}^{(t)} + (1-\beta)\tilde{\mathbf{Y}}_{\text{cls}}^{\text{meta}}$
Output: $\tilde{\mathbf{Y}}_{\text{cls}}^{(t+1)}$

Algorithm 2 PU Pre-training

Input: Total Dataset $\mathcal{D} = \mathcal{D}_L + \mathcal{D}_U$, Validation Labeled Dataset $\mathcal{D}_L^{\text{val}}$, Encoders $\phi_{\text{Ab}}, \phi_{\text{Ag}}$, Classifier f_{cls}, Warm-up epochs T_{warm}, Momentum β
Initialize parameters $\theta_\phi, \theta_{\text{cls}}$, pseudo-labels $\tilde{\mathbf{Y}}_{\text{cls}}$, and cluster-aware loss $\mathcal{L}_{\text{Cluster}} \leftarrow 0$
for epoch $t = 1$ **to** MaxEpochs **do**
  Sample mini-batch $\mathcal{B} = \mathcal{B}_L \cup \mathcal{B}_U$
  Generate multi-view representations $\mathbf{e}, \mathbf{e}', \mathbf{e}''$
  $\mathcal{L}_{\text{Ins}} \leftarrow$ Instance Contrastive Loss (Eq. 1)
  if $t > T_{\text{warm}}$ **then**
    // Bi-Level Meta-Optimization
    Refine $\tilde{\mathbf{Y}}_{\text{cls}}$ using **algorithm 1**
    // Cluster-aware Optimization
    Identify positive set $\mathcal{P}$ via prototype alignment
    $\mathcal{L}_{\text{Cluster}} \leftarrow$ Cluster-aware Loss (Eq. 2)
  end if
  $\mathcal{L}_{\text{CE}} \leftarrow$ Cross-Entropy with targets $\tilde{\mathbf{Y}}_{\text{cls}}$
  Update $\theta_\phi, \theta_{\text{cls}} \leftarrow \text{Optimizer}(\nabla(\mathcal{L}_{\text{CE}} + \mathcal{L}_{\text{Ins}} + \mathcal{L}_{\text{Cluster}}))$
end for
Output: Pre-trained encoders $\phi_{\text{Ab}}, \phi_{\text{Ag}}$

where α is the meta-learning rate. Subsequently, we evaluate the virtually updated model θ' on a clean validation batch $\mathcal{D}_L^{\text{val}}$ (with the labels of $\tilde{\mathbf{Y}}_{\text{cls}}^{\text{val}}$) to seek the optimal perturbation $\mathbf{\Delta}^*$ that minimizes the validation loss:

$$\mathbf{\Delta}^* = \arg\min_{\mathbf{\Delta}} \mathcal{L}_{\text{CE}}(f_{\text{cls}}(\mathbf{e}_{\theta'}), \tilde{\mathbf{Y}}_{\text{cls}}^{\text{val}}). \tag{S6}$$

Here, $\mathbf{e}_{\theta'}$ indicates the validation representations, computed using the updated encoder parameters within θ'. Guided by this optimization, we derive refined discrete pseudo-labels $\tilde{\mathbf{Y}}_{\text{cls}}^{\text{meta}} = \text{OneHot}(\arg\max(\tilde{\mathbf{Y}}_{\text{cls}} + \mathbf{\Delta}^*))$. Finally, to stabilize training, we update the targets for the subsequent epoch using an Exponential Moving Average (EMA) with the momentum coefficient of β (Hu et al., 2023):

$$\tilde{\mathbf{Y}}_{\text{cls}}^{(t+1)} = \beta \tilde{\mathbf{Y}}_{\text{cls}}^{(t)} + (1-\beta)\tilde{\mathbf{Y}}_{\text{cls}}^{\text{meta}}. \tag{S7}$$

The complete bi-level meta-optimization procedure is summarized in algorithm 1 and the pre-training phase is detailed in algorithm 2. The selection for hyperparameters are summarized in table S4.

S5 Details for Ranking Module

The Ranking Module first projects the input matrix $\mathbf{E}_{\mathcal{T}} \in \mathbb{R}^{K \times 2d_{\text{out}}}$ to a hidden dimension d_r, yielding the initial features $\mathbf{Z}^{(0)} \in \mathbb{R}^{K \times d_r}$. Then, N_r layers of ISAB are applied with M learnable inducing points $\mathbf{I} \in \mathbb{R}^{M \times d_r}$ for robust context modeling. The update rule for the l-th layer is defined as:

$$\begin{aligned} \mathbf{H}_{\text{ind}} &= \text{MHA}(\mathbf{I}, \mathbf{Z}^{(l-1)}, \mathbf{Z}^{(l-1)}) + \mathbf{I}, \\ \mathbf{Z}^{(l)} &= \text{rFF}(\text{MHA}(\mathbf{Z}^{(l-1)}, \mathbf{H}_{\text{ind}}, \mathbf{H}_{\text{ind}}) + \mathbf{Z}^{(l-1)}), \end{aligned} \tag{S8}$$

where $\text{MHA}(\cdot)$ denotes Multi-Head Attention and $\text{rFF}(\cdot)$ is a row-wise feed-forward network. The inducing points $\mathbf{I}$ serve as a global workspace that aggregates information from the input set $\mathbf{Z}^{(l-1)}$ into $\mathbf{H}_{\text{ind}}$, and then distributes the

Algorithm 3 MHSA Ranking

Input: Tuple set $\mathcal{T}$ (constructed via Homologous Sampling)
Input: Pre-trained Encoders $\phi_{\text{Ab}}, \phi_{\text{Ag}}$, Set Transformer f_{rank}
Hyperparameters: Learning rate η, Batch size B
Initialize ranking module parameters θ_{rank}
for epoch $t = 1$ **to** MaxEpochs **do**
  for each mini-batch of tuples $\{\mathcal{T}\}_B$ **do**
    // Forward pass
    Extract embeddings $\mathbf{E}_{\mathcal{T}}$ for tuples via $\phi_{\text{Ab}}, \phi_{\text{Ag}}$
    Predict ranking scores $\mathbf{r} = f_{\text{rank}}(\mathbf{E}_{\mathcal{T}})$
    // Optimization
    Compute $\mathcal{L}_{\text{ListMLE}}$ (Eq. 6)
    Update $\theta_\phi, \theta_{\text{rank}} \leftarrow \text{Optimizer}(\nabla \mathcal{L}_{\text{ListMLE}})$
  end for
end for

refined context back to update the features (Lee et al., 2019). Detailed implementations of the ranking module and hyperparameter configurations are provided in algorithm 3 and table S4, respectively.

Table S4: Hyperparameter settings for AbLWR. The model is trained in two stages: PU Pre-training (left) and MHSA Ranking (right).

**PU Pre-training**		**MHSA Ranking**	
**Parameter**	**Value**	**Parameter**	**Value**
Optimizer	SGD	Optimizer	Adam
Max Epochs	400	Max Epochs	50
Warm-up Epochs	20	Batch Size	16
Batch Size	64	Learning Rate	0.001
Learning Rate	0.001	Weight Decay	0.3
Meta LR	0.001	Inducing Points (M)	5
Weight Decay	1×10^{-4}	Attention Heads	4
Momentum	0.9	Dropout Rate	0.2
Temperature (τ)	0.07	-	-
ρ Range	$[0.8, 0.95]$	-	-

S6 Training Details

S6.1 Baseline Models

We benchmark AbLWR against seven representative baseline methods. Specifically, AbRank adopts a pairwise approach, explicitly learning to contrast the affinity of two Ab-Ag pairs. Conversely, GraphDTA, MINT, AntiBERTy, FoldX, ANTIPASTI, and LambdaMART functions are pointwise regression models, aiming to predict or simulate the absolute binding affinity values for independent Ab-Ag pair.

- **AbRank (Liu et al., 2025):** A metric-learning framework for Ab-Ag affinity ranking. To adapt it for our specific evaluation, we retrained the model on the datasets provided by the authors, strictly removing any test set leakage (*balanced-train-swapped.csv* for Random Split, *hard-ag-train-swapped.csv* for Ag-based Split, and *hard-ab-train-swapped.csv* for Ab-based Split). We utilized the official implementation from GitHub[1] with the standard configuration file *train-abrank.yaml* for all experiments.

- **GraphDTA (Nguyen et al., 2021):** A GNN-based model originally designed to represent molecular interactions as graphs for binding affinity prediction. In our experiments, we adjusted the maximum epochs to 50 and the batch size to 64 due to computational constraints, while keeping all other hyperparameters consistent with the original paper. We adopted the architecture directly from its official repository[2].

[1] `https://github.com/biochunan/AbRank-WALLE-Affinity`
[2] `https://github.com/thinng/GraphDTA`

- **MINT (Ullanat et al., 2026):** A PLM-based method designed to model protein interactions in a contextualized manner. We employed MINT as a feature extractor to obtain embeddings for Ab-Ag pairs, followed by a simple MLP to predict binding affinity. We used the pre-trained checkpoint *bernett_mlp.pth* available on Hugging Face[3] with the configuration file *esm2_t33_650M_UR50D.json*. The code was adapted from the official repository[4].
- **AntiBERTy (Ruffolo et al., 2021):** An antibody-specific transformer pre-trained on 558M natural antibody sequences. Similar to our MINT setup, we utilized the `AntiBERTyRunner` library to extract embeddings for Ab-Ag pairs, which were then fed into an MLP for binding affinity prediction optimized by MSE loss. We set the hyperparameters (Max Epochs=50, Learning Rate=1×10^{-3}) to match our proposed method where applicable. The implementation was based on the official repository[5].
- **LambdaMART (Burges, 2010):** A classic Learning-to-Rank algorithm based on gradient boosted decision trees. We implemented the model using the LightGBM library with the `lambdarank` objective to directly optimize Mean Reciprocal Rank (MRR) and used AntiBERTy embeddings as input features. The model was trained for 100 boosting rounds with a learning rate of 1×10^{-3}, a maximum depth of 4, and L1/L2 regularization of 0.1.
- **FoldX (Schymkowitz et al., 2005):** A physics-based method that estimates protein stability ($\Delta\Delta G$) based on 3D structural information. Since our dataset lacks experimental complex structures, we utilized HDOCK (Yan et al., 2020), a protein-protein docking web server, to generate the binding conformations for each Ab-Ag pair before computing $\Delta\Delta G$.
- **ANTIPASTI (Michalewicz et al., 2024):** A CNN-based model that utilizes normal mode correlation maps derived from elastic network models to predict affinity. Similar to the FoldX setup, we prepared the binding modes using HDOCK prior to prediction. Our implementation followed the tutorial *[Tutorial] Predicting affinity using ANTIPASTI.ipynb* provided in the official GitHub repository[6].

S6.2 HDOCK-based structure-generation protocol

FoldX, ANTIPASTI and PBEE require complex structures for scoring. Since experimentally determined antibody-antigen complex structures were not available for all evaluated pairs, we generated the required binding conformations using HDOCK. The same HDOCK-generated complex structure was used for all of these three baseline methods. Antibody monomer structures were predicted using IgFold from the Chothia-defined heavy- and light-chain variable-region sequences. Antigen monomer structures were predicted from their amino-acid sequences using ESMFold. The antibody and antigen PDB files were then supplied as the first and second HDOCK inputs, respectively:

```
./hdock antibody.pdb antigen.pdb -out Hdock.out
```

No additional binding-site restraints were specified. The default HDOCKlite docking and scoring configuration was used. Docked complex structures were generated using:

```
./createpl Hdock.out model_1.pdb -nmax 1 -complex -models
```

Only the top-ranked pose according to the internal HDOCK score was retained. The resulting complex was saved as `AbAg_{complex ID}_Complex.pdb` and used as the common structural input for all three baselines.

For FoldX, the HDOCK-generated complex was evaluated using the `AnalyseComplex` command, with the antibody and antigen chains specified as the two interacting partners. For ANTIPASTI and PBEE, the same HDOCK coordinates were used as input without selecting any alternative docking pose.

S6.3 Evaluation Metrics

In our experiments, we evaluate the performance on a test set consisting of N query lists. For the i-th list $\mathcal{T}^{(i)}$, let $\mathbf{y}^{(i)} = [y_1^{(i)}, \dots, y_K^{(i)}]^\top$ denote the ground-truth binding affinities, and $\mathbf{r}^{(i)} = [r_1^{(i)}, \dots, r_K^{(i)}]^\top$ denote the predicted ranking scores, where $K = 5$. The metrics are calculated for each list and then averaged across the entire test set. The detailed definitions of the five metrics are as follows:

- **FRA:** Measuring the percentage of lists where the predicted ranking order perfectly matches the ground-truth order.

$$\text{FRA} = \frac{1}{N} \sum_{i=1}^{N} \mathbb{I}\left(\text{rank}(\mathbf{r}^{(i)}) = \text{rank}(\mathbf{y}^{(i)})\right)$$

where $\mathbb{I}(\cdot)$ is the indicator function, and $\text{rank}(\mathbf{v})$ returns the permutation indices that sort the vector $\mathbf{v}$.

[3]`https://huggingface.co/varunullanat2012/mint`
[4]`https://github.com/VarunUllanat/mint`
[5]`https://github.com/jeffreyruffolo/AntiBERTy`
[6]`https://github.com/kevinmicha/ANTIPASTI`

- **$K\tau$:** Evaluating the ordinal association between the predicted score vector $\mathbf{r}$ and the ground-truth vector $\mathbf{y}$.

$$\tau = \frac{1}{N}\sum_{i=1}^{N}\frac{CP^{(i)} - DP^{(i)}}{\frac{1}{2}K(K-1)}$$

  where CP and DP denote the number of concordant pairs and discordant pairs within the list, respectively. A value of 1 implies a perfect match, while -1 implies a perfectly reversed order.

- **PRA:** Quantifiying the fraction of correctly ordered pairs within a list to measure the model's ability to satisfy pairwise constraints.

$$\text{PRA} = \frac{1}{N}\sum_{i=1}^{N}\left[\frac{1}{\binom{K}{2}}\sum_{u=1}^{K-1}\sum_{v=u+1}^{K}\mathbb{I}\left((r_u^{(i)} - r_v^{(i)})(y_u^{(i)} - y_v^{(i)}) > 0\right)\right]$$

  where $\binom{K}{2}$ denote the number of possible unique pairs within a list of size K.

- **PAU:** Quantifying the fraction of correctly ordered pairs within each list to measure the model's ability to satisfy pairwise constraints by reformulating the ranking problem as a binary classification task, and computing the Area Under the ROC Curve (AUC) over predicted score and ground-truth preferences.

- **P@1:** Measuring the proportion of lists where the highest-affinity candidate is correctly identified as the top-1 prediction, reflecting the model's practical utility in antibody discovery.

$$\text{P@1} = \frac{1}{N}\sum_{i=1}^{N}\mathbb{I}\left(\arg\min(\mathbf{r}^{(i)}) = \arg\min(\mathbf{y}^{(i)})\right)$$

S6.4 Computational Requirements

We further summarized the computational requirements of AbLWR, including the number of trainable parameters, training time, inference time, and peak GPU memory usage. All experiments were conducted on a single NVIDIA GeForce RTX 3090 GPU with 24 GB memory.

AbLWR consists of two training stages: PU pretraining and listwise fine-tuning. The PU pretraining model contains 0.30M trainable parameters and was trained for 400 epochs. The average training-loop duration was approximately 7.72 minutes per epoch, resulting in a total training time of about 51.50 hours. The listwise fine-tuning model contains 9.34M trainable parameters and was trained for 50 epochs, requiring approximately 14.76 hours in total. A complete inference pass over the test set required approximately 4.70 minutes. The estimated peak GPU memory consumption was approximately 8.39 GiB during PU pretraining and 4.74 GiB during listwise fine-tuning. The computational cost is summarized in Supplementary Table S5.

Table S5: Computational cost of AbLWR.

Metric	PU pretraining	Listwise fine-tuning
Trainable parameters (M)	0.30	9.34
Training time (h)	51.50	14.76
Inference time (min)	–	4.70
Peak GPU memory (GiB)	8.39	4.74

S7 Sensitivity Analysis of List Sampling Hyperparameters

S7.1 Impact of list size K and minimum margin y_{cutoff}

We evaluate the model's sensitivity to the list size ($K \in [3, 10]$) and the minimum margin ($y_{\text{cutoff}} \in \{0.1, 0.5, 1.0, 2.0\}$), bounded by the capacity of our available hardware (a single NVIDIA RTX 3090 GPU with 24GB VRAM). The detailed results are presented in table S6, where the numbers in parentheses denote the count of valid lists across the train, validation, and test splits.

The model demonstrates robust performance across various K values and y_{cutoff} thresholds. As K increases, a slight performance degradation is observed. This is primarily attributed to the exponential expansion of the permutation space ($K!$) and a reduction in the number of valid training lists that satisfy the sampling criteria. Despite this, Kendall's τ and P@1 remain relatively stable even at larger K values, which aligns perfectly with the primary objective of

Table S6: Sensitivity analysis of list sampling hyperparameters for K and y_{cutoff}.

**Parameter**	**Value**	**Kendall's τ**	**P@1 (%)**
List Size (K)	3 (105688/21870/21829)	0.59	**71.53**
	4 (84969/20123/19992)	0.53	56.53
	5 (56878/13095/13030)	**0.60**	57.73
	6 (60116/15290/15378)	0.49	46.35
	7 (51803/14007/14063)	0.51	44.99
	8 (45386/13192/13215)	0.51	42.05
	9 (40355/12632/12643)	0.49	42.13
	10 (36314/12042/12026)	0.46	41.64
Margin (y_{cutoff})	0.1 (62380/14661/14533)	0.58	56.88
	0.5 (56878/13095/13030)	**0.60**	**57.73**
	1.0 (42001/9926/9827)	0.58	56.07
	2.0 (11401/3196/3194)	0.49	50.30

practical wet-lab screening: accurately identifying the Top-1 binder. Our default setting of $K = 5$ and $y_{\text{cutoff}} = 0.5$ optimally balances contextual learning capacity and computational efficiency. Specifically, a smaller K reduces the task to mere pairwise comparisons, whereas a larger K significantly exacerbates VRAM consumption and optimization difficulty.

Currently, applying the model to larger candidate pools requires retraining for a specific K. While this introduces computational overhead, it remains highly feasible in practice; the total training and evaluation pipeline takes approximately 12 hours on an RTX 3090 GPU. Given that wet-lab screening typically costs tens of thousands of dollars and spans several weeks, this retraining time is a negligible trade-off for real-world deployments. To further enhance scalability, decoupling the model architecture from a fixed K is a promising direction. In future work, we plan to explore a dynamic Elo rating-based framework, treating affinity ranking as "matches" between candidate subsets to iteratively update global Elo scores. This approach would enable the model to handle arbitrary pool sizes without retraining, while effectively mitigating the performance decline observed at larger K values.

S7.2 Performance comparison across different sequence identity

We further evaluated our model's performance on test samples filtered by much stricter sequence identity thresholds (SeqIdent) of < 0.8, < 0.3 and < 0.2 in Ag-based and Ab-based (Cluster 5) Split. The SeqIdent is computed by BLOSUM62 normalized similarity, which comprehensively accounts for the evolutionary and physicochemical conservatism of amino acid substitutions.

Table S7: Performance comparison across different sequence identity thresholds.

**Split**	**SeqIdent**	**Kendall's τ**	**P@1 (%)**
Ag-based	< 0.8	0.49	53.50
	< 0.3	0.51	50.10
	< 0.2	0.51	50.10
Ab-based	< 0.8	0.42	50.00
	< 0.3	0.37	46.32
	< 0.2	0.38	47.29

As shown in the table, the model demonstrates remarkable robustness under two different split scenarios. The Ab-based Split presents a significantly harder challenge due to the extreme diversity of antibody CDRs. Specifically, a SeqIdent < 0.3 implies generalizing across entirely different antibody families. Despite this extremely difficult zero-shot scenario, the metrics most critical for practical screening (Kendall's τ and P@1) experience only a mild decline and remain acceptable.

S7.3 Robustness to Random List Sampling

A central step in AbLWR is the construction of candidate lists for listwise ranking. Since the candidate lists are sampled during both training and evaluation, different random seeds may lead to different combinations of antibody-antigen pairs within the constructed lists. To evaluate the robustness of AbLWR to this randomness, we repeated the full

training and evaluation pipeline using three different random seeds. For each seed, the training and test candidate lists were reconstructed independently, and the model was trained from scratch under the same experimental setting. We then evaluated the trained models using the same ranking metrics as in the main experiments.

As shown in Table S8, the performance of AbLWR remains stable across different random seeds. The standard deviations are small for all evaluation metrics, with FRA varying by only 0.27%, PRA by 0.26%, PAU by 0.24%, and P@1 by 0.41%. These results indicate that the reported performance is not highly dependent on a particular sampled list set.

Table S8: Robustness analysis of AbLWR under different random seeds. For each seed, the candidate lists were reconstructed, and the full training and evaluation pipeline was repeated from scratch.

**Random Seed**	**FRA (%)**	K_τ	**PRA (%)**	**PAU (%)**	**P@1 (%)**
Seed 1	21.28	0.60	79.96	80.14	57.73
Seed 2	21.70	0.61	80.39	80.55	58.22
Seed 3	21.78	0.61	80.41	80.57	58.54
Mean ± Std	21.58 ± 0.27	0.61 ± 0.00	80.25 ± 0.26	80.42 ± 0.24	58.16 ± 0.41

S8 Additional Ablation Studies

To further examine the contributions of the main design choices in AbLWR, we conduct two additional ablation studies. First, we compare the induced self-attention block (ISAB) with a standard self-attention block (SAB) to evaluate whether the inducing-point formulation provides benefits beyond generic self-attention. Second, we replace the listwise objective with a pairwise ranking loss while keeping the remaining AbLWR framework unchanged, in order to more directly assess the effect of the ranking objective. The results are summarized in Table S9.

Table S9: Additional ablation studies on the set attention module and ranking objective.

**Model Variant**	**FRA (%)**	**Kendall's tau**	**PRA (%)**	**PAU (%)**	**P@1 (%)**
**AbLWR (Ours)**	**21.28**	**0.60**	**79.96**	**80.14**	**57.73**
w/ SAB	13.37	0.53	76.31	76.48	53.08
w/ Pairwise Loss	11.44	0.52	75.98	76.15	50.79

Effect of the induced self-attention block. To disentangle the contribution of the inducing-point formulation from that of self-attention in general, we replace the ISAB module in AbLWR with a standard SAB (Lee et al., 2019) while keeping the other components unchanged. Table S9 shows that replacing ISAB with SAB leads to consistent performance degradation across all evaluation metrics. FRA decreases from 21.28% to 13.37%, and Kendall's tau decreases from 0.60 to 0.53. PRA and PAU also decrease by 3.65 and 3.66 percentage points, respectively, while P@1 drops from 57.73% to 53.08%. These results suggest that the benefit of ISAB stems not merely from introducing a generic self-attention mechanism but also from improved context modeling. The inducing points in ISAB can be viewed as learnable global prototypes that aggregate set-level information and mediate interactions among candidate antibodies. This introduces a structured bottleneck that encourages each element to attend to shared global context patterns.

Effect of the listwise ranking objective. The proposed AbLWR formulates affinity prediction as a listwise ranking problem and optimizes the model using the ListMLE objective. To directly examine the contribution of the listwise objective, we conduct an ablation study by replacing it with a pairwise ranking loss while keeping the rest of the AbLWR framework unchanged. Specifically, we adopt the same pairwise loss function used in AbRank (Liu et al., 2025), so that the main difference between this ablated variant and the full AbLWR model lies in the ranking objective itself. Table S9 shows that the ListMLE-based objective performs consistently better than the pairwise ranking loss within the same AbLWR framework. Replacing the listwise loss with the pairwise loss reduces FRA from 21.28% to 11.44%, Kendall's tau from 0.60 to 0.52, and P@1 from 57.73% to 50.79%. PRA and PAU also decrease by 3.98 and 3.99 percentage points, respectively. This trend suggests that the listwise objective is better aligned with the goal of ranking all candidates within a set, whereas the pairwise loss only optimizes local pairwise preferences.

S9 Details for Case Studies

S9.1 Influenza Viruses

Table S10: **In vitro neutralization IC_{50} values (μg/mL) and corresponding PDB codes.** Data are adapted from Momont et al. (Momont et al., 2023). Lower IC_{50} values indicate higher potency. Entries marked as '> 50' highlight the loss of neutralization efficacy (immune escape). '–' denotes that the crystal structure is unavailable.

			In vitro neutralization IC_{50} (μg/mL)					
			FNI9		FNI17		FNI19	
**Strain**	**Abbr.**	**Group**	IC_{50}	PDB	IC_{50}	PDB	IC_{50}	PDB
A/Darwin/6/2021	A/DW/2021	H3N2	1.128	–	> 50	–	2.790	–
A/HongKong/2671/2019	A/HK/2019	H3N2	0.073	8G3P	5.068	–	0.190	8G40
A/Singapore/INFIMH-16-0019/2016	A/SA/2016	H3N2	0.162	–	> 50	–	0.916	–
B/Massachusetts/2/2012	B/MA/2012	Yamagata	0.204	–	0.125	–	0.472	8G3Z
B/Phuket/3073/2013	B/PK/2013	Yamagata	0.193	–	0.117	–	0.222	–

To validate the practical utility of AbLWR in distinguishing antibody efficacy across evolving viral strains, we selected three monoclonal antibodies (mAbs)—FNI9, FNI19, and FNI17—which have demonstrated broad-spectrum binding against a diverse panel of neuraminidases (NAs) from seasonal and zoonotic influenza A (IAV) and B (IBV) viruses (Momont et al., 2023). Among these, FNI17 presents a particularly instructive challenge: while it effectively neutralizes historical H3N2 strains, its potency is markedly reduced against variants emerging after 2015 due to antigenic drift. In our analysis, we constructed a specific evaluation set comprising 15 Ab-Ag pairs (involving three antibodies and five antigens, as detailed in table S10). By exhaustively combining these pairs, we included 3,003 unique lists to rigorously test the ranking capabilities of our model. Detailed quantitative results on ranking fidelity are provided in table S11 and fig. S3, which summarizes the success rates of AbLWR across different Kendall's τ cutoffs.

Table S11: **Quantitative evaluation of ranking fidelity for AbLWR on influenza virus and human IL-33 datasets.** The values represent the percentage of test cases where the $K\tau$ exceeds the specified thresholds $(0.2, 0.4, 0.6)$.

**Metric**	**Influenza Virus**	**Human IL-33**
Kendall's $\tau > 0.2$	67.83%	45.32%
Kendall's $\tau > 0.4$	43.32%	27.43%
Kendall's $\tau > 0.6$	18.02%	13.26%

Then, we adopted the same sampling methodology employed for the Ab-based Split (as detailed in section S2.3 with $y_{\text{cutoff}} = 0.1$) to construct the evaluation lists presented in fig. 3 (AbLWR) and fig. S4 (representative data driven baselines). As shown in fig. S4, among these baselines, only AbRank demonstrates a meaningful positive correlation with an average Kendall's τ of 0.42. Conversely, the other three models—AntiBERTy, GraphDTA, and ANTIPASTI—struggle to distinguish candidates within this narrow affinity range. In comparison, our proposed AbLWR outperforms all baselines, achieving the highest average Kendall's τ of 0.47. Visually, the heatmap for AbLWR demonstrates a more distinct boundary between high- and low-affinity candidates compared to AbRank.

We further compared the ranking performance on the FNI17 antibody mechanism, as visualized in fig. S5. Consistent with the results on the sampled lists, AntiBERTy, GraphDTA, and ANTIPASTI failed to capture the correct ranking relationship (lines crossing significantly in fig. S5 (a), (c), (d)). While AbRank (fig. S5 (b)) captures the general ranking trend, this global alignment masks a critical deficiency in practical screening scenarios. As shown in fig. 4 (a), AbLWR successfully identified and correctly ordered the top-2 high-affinity strains, placing the ground truth Rank 1 (B/PK/2013) and Rank 2 (B/MA/2012) candidates in their exact predicted positions. In contrast, AbRank failed to distinguish the precise order of these top-tier binders. This demonstrates that our method possesses superior sensitivity in the high-affinity region, which is the most decisive factor for prioritizing lead candidates in antibody discovery.

S9.2 Human IL-33

To further evaluate the generalization capability of AbLWR, particularly on antibody architectures distinct from the training distribution, we conducted an additional case study on human Interleukin-33 (IL-33). We utilized the dataset from Wang et al.(Wang et al., 2025), which describes the optimization of antibody I7 against human IL-33. Crucially, I7 is a single-chain antibody, representing a significant structural deviation from the paired-chain antibodies typically found in standard training datasets. The dataset consists of 20 antibody variants (the wild type and 19

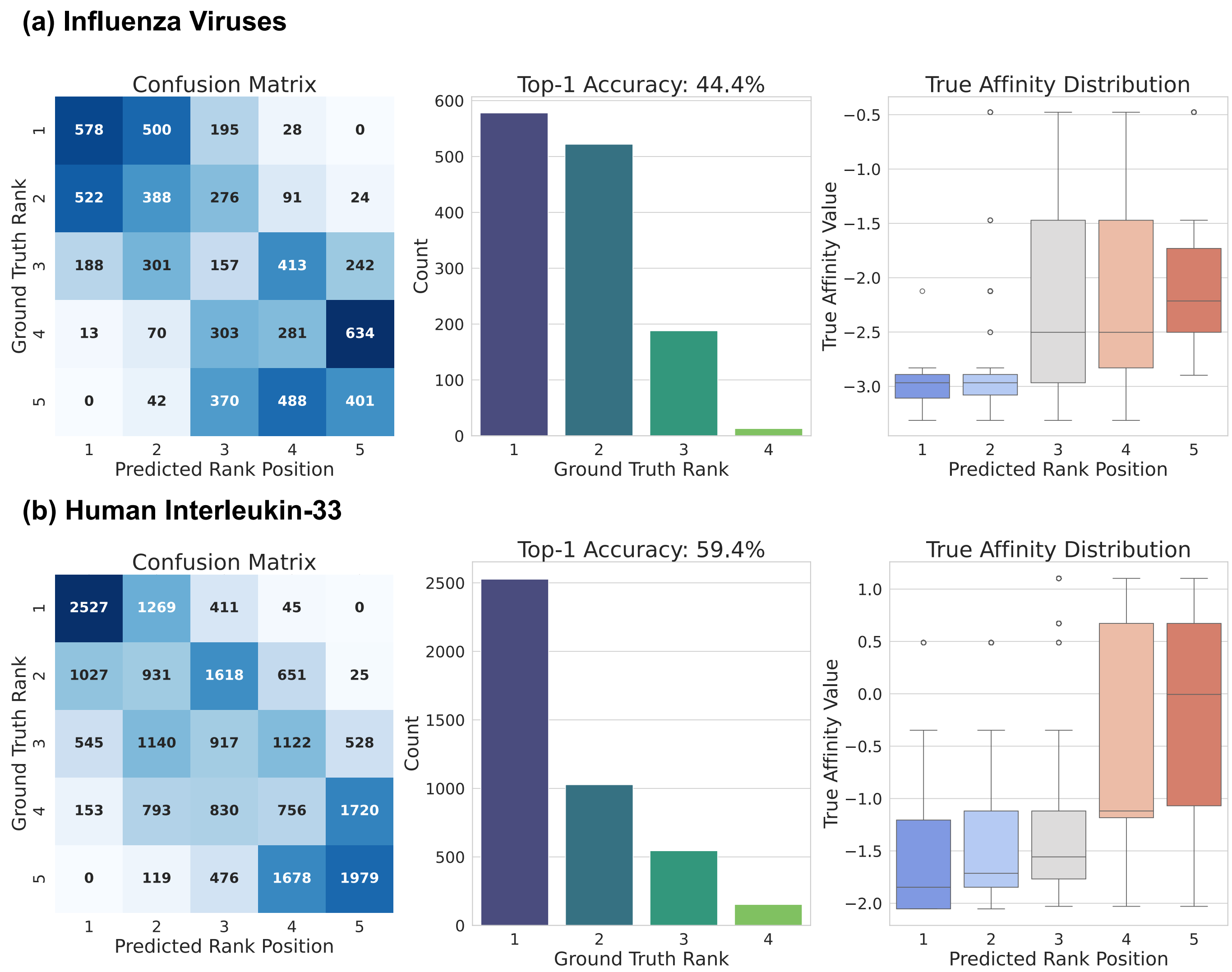

Figure S3: **Detailed visualization of ranking performance on (a) influenza viruses and (b) human IL-33.** The comparison includes (left) Confusion Matrices between predicted and ground truth ranks, (middle) Distribution of Ground Truth Ranks for candidates predicted as Top-1, and (right) Boxplots showing the true affinity value distribution across predicted rank positions.

Table S12: **Experimental EC_{50} values (nM) of the I7 single-chain antibody and its variants against Human IL-33 (Wang et al., 2025).** The dataset comprises the wild-type (I7-WT) and 19 generated variants, where lower values indicate stronger binding affinity.

Cand.	EC_{50}	Cand.	EC_{50}	Cand.	EC_{50}	Cand.	EC_{50}
I7-WT	11.41	I7-M5	1898	I7-M10	1.712	I7-M15	705.3
I7-M1	2.561	I7-M6	462.2	I7-M11	4.16	I7-M16	1.402
I7-M2	37.15	I7-M7	2.898	I7-M12	16.2	I7-M17	1.326
I7-M3	148.1	I7-M8	5961	I7-M13	9.857	I7-M19	2.134
I7-M4	12.79	I7-M9	9.845	I7-M14	67.34	I7-M20	9.352

generated variants) with experimentally measured binding affinities (see table S12). To derive a global ranking for the 20 candidates, we aggregated local predictions from our list-wise model and averaged across all occurrences. The final ranking was determined by sorting these mean scores.

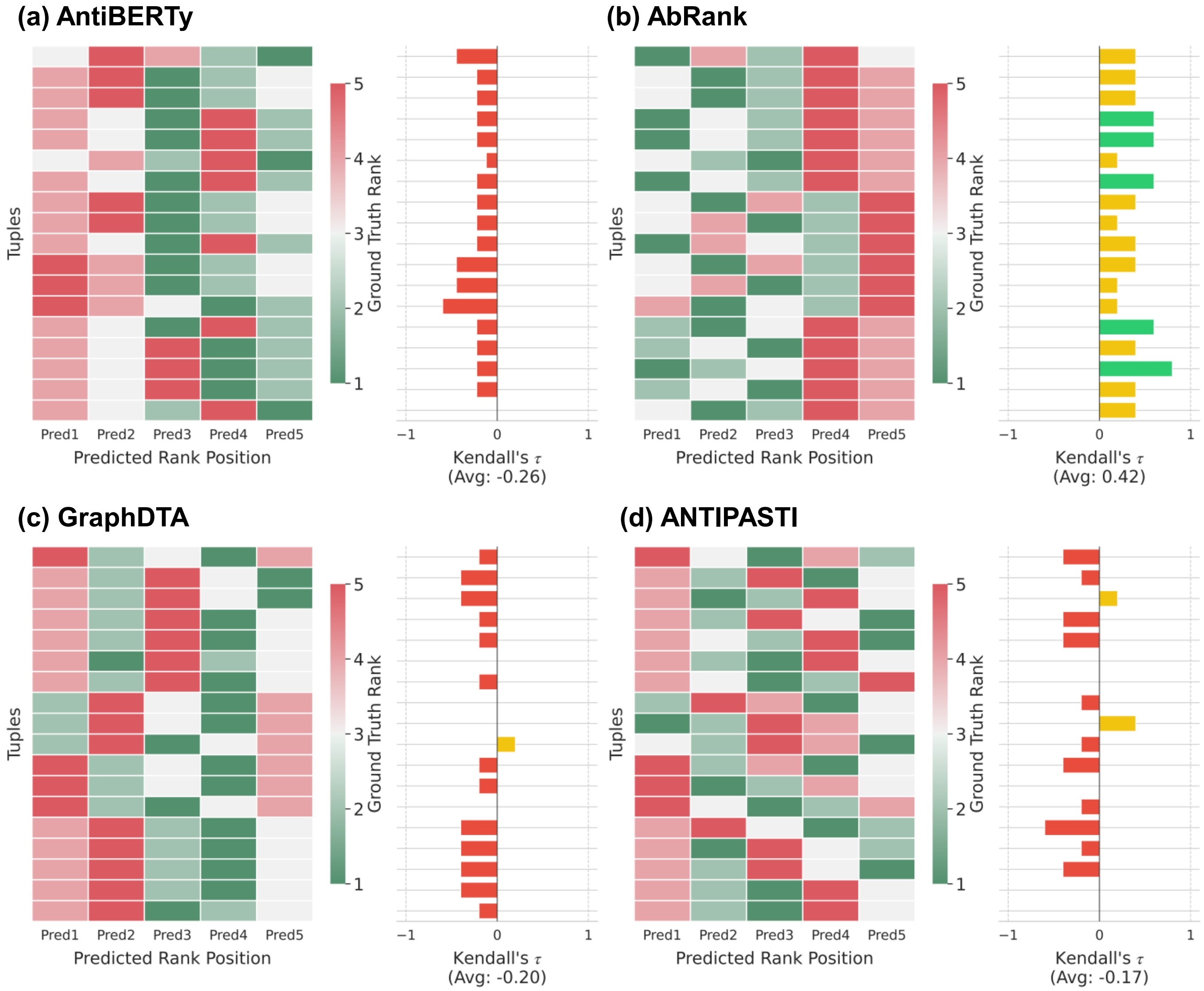

Figure S4: **Visualization of ranking predictions on 18 sampled lists by four representative data driven baseline models.** (a) AntiBERTy (sequence-based); (b) AbRank (structure-based pairwise ranking); (c) GraphDTA (structure-based); and (d) ANTIPASTI (complex-based).

S10 Sequence Similarity and Leakage Analysis for the Case Studies

To verify that the case-study data were not present in the training set, we performed a quantitative sequence-similarity and leakage analysis for all antibodies and antigens used in the Influenza/FNI17 and Human IL-33/I7 case studies against the exact training split. Throughout this analysis, "sequence similarity" was quantified as percent sequence identity, calculated as the number of identical residue pairs divided by the total alignment length, including internal gaps.

S10.1 Sequence alignment and analysis protocol

All sequence alignments were performed using BLOSUM62 with affine gap penalties (Henikoff and Henikoff, 1992; Gotoh, 1982). Specifically, antibody variable regions were globally aligned because the heavy-chain and light-chain variable domains are homologous structural units of comparable lengths (Needleman and Wunsch, 1970). Antigens were locally aligned because the training data may contain full-length proteins, isolated domains, or experimentally resolved fragments of different lengths (Smith et al., 1981). To avoid interpreting short conserved motifs as close protein-level matches, we retained only antigen alignments covering at least 80.00% of the case-study antigen sequence. This

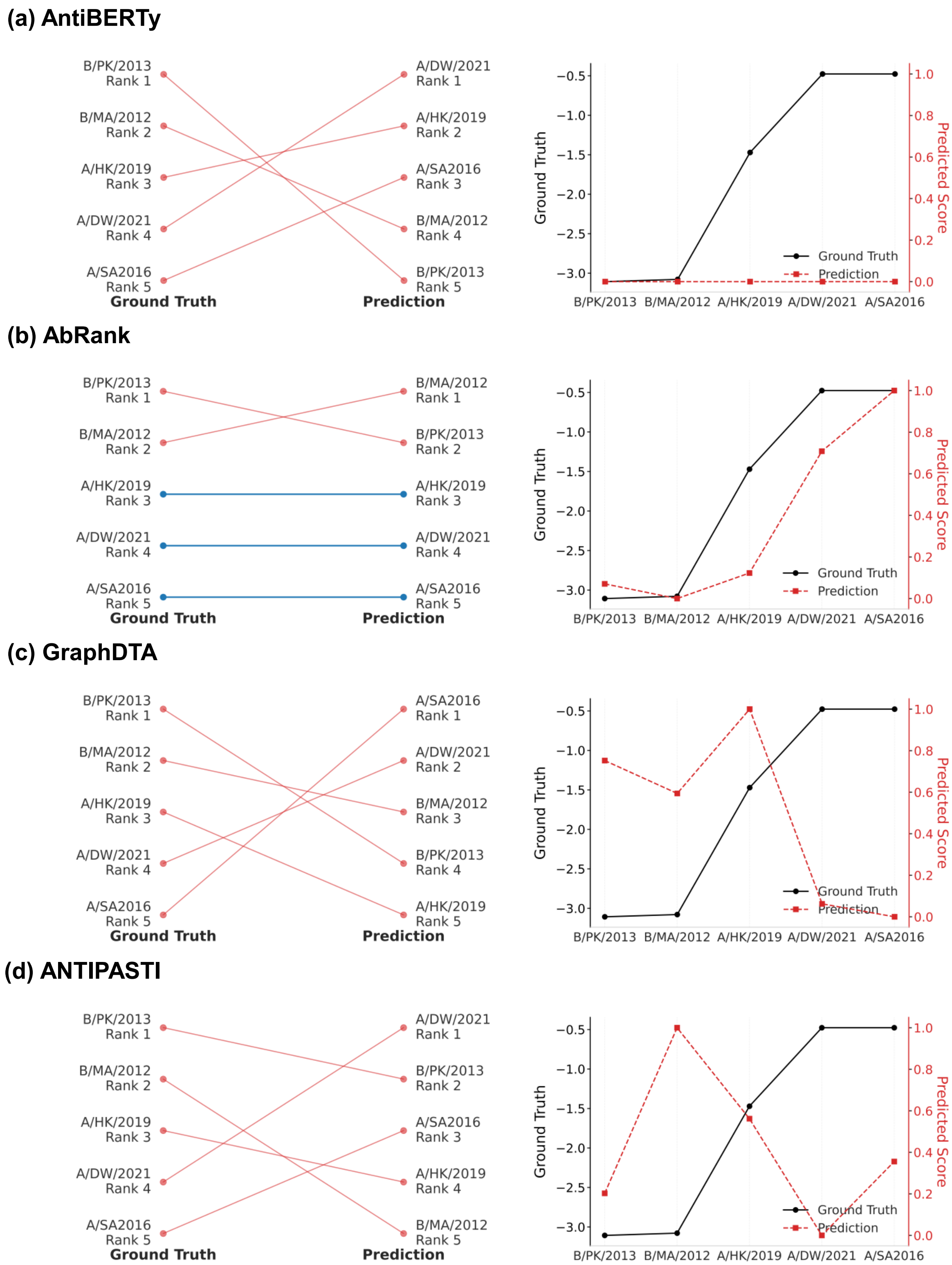

Figure S5: **Analysis of FNI17 antibody predictions by four baselines.** Models: (a) AntiBERTy (sequence-based); (b) AbRank (structure-based pairwise ranking); (c) GraphDTA (structure-based); (d) ANTIPASTI (complex-based).

requirement was used as an operational query-coverage filter for local alignments, consistent with the general practice of considering both sequence identity and alignment coverage when interpreting sequence-similarity searches (Fu et al., 2012).

To rigorously evaluate potential data leakage, we assessed similarities at both the individual protein and complex levels. First, at the individual-protein level, we separately compared each case-study antibody and antigen against the training set to detect any high similarity or exact overlap. Second, because AbLWR predicts specific antibody–antigen interactions rather than isolated proteins, we examined joint similarity and complete overlap at the pair level. Specifically, we defined pair-level joint similarity as the smaller of the antibody and antigen similarities computed against the same training complex. This conservative definition prevents high similarity in only one component from inflating the apparent similarity of the complete antibody–antigen complex (Park and Marcotte, 2012). Together, these complementary approaches allow us to comprehensively quantify the extent of data leakage across different potential scenarios.

Table S13: **Case-study protein similarity to the training set.** Antibody rows report HCDR3 similarity, whereas antigen rows report protein-level similarity after applying the query-coverage filter. × indicates that no exact sequence overlap was found in the training set.

Query Protein	Exact Overlap	Protein-level Similarity	HCDR3 Similarity	Query Coverage
Influenza/FNI17				
FNI17 antibody	×	N/A	15.79%	100.00%
B/PK/2013 antigen	×	32.28%	N/A	93.08%
B/MA/2012 antigen	×	32.10%	N/A	99.23%
A/HK/2019 antigen	×	91.45%	N/A	99.48%
A/DW/2021 antigen	×	21.60%	N/A	89.14%
A/SA/2016 antigen	×	77.09%	N/A	99.54%
IL-33/I7				
I7-WT and 19 variants	×	N/A	36.36–50.00%	100.00%
IL-33 antigen	×	25.70%	N/A	91.82%

S10.2 Individual-protein similarity analysis

The resulting individual-protein similarities are summarized in Table S13. For the antibody component, no exact case-study antibody sequence was found in the training set. Because antibody specificity is strongly influenced by the heavy-chain complementarity-determining region 3 (HCDR3), we focused on HCDR3 similarity when comparing the case-study antibodies with the training antibodies. FNI17 showed a maximum HCDR3 similarity of only 15.79%, indicating substantial divergence in this highly variable and binding-relevant region. For the I7 affinity-maturation series, the maximum HCDR3 similarities of I7-WT and its 19 variants to the training antibodies ranged from 36.36% to 50.00%. These results indicate that the case-study antibodies were not exact training-set matches and did not share highly similar HCDR3 sequences with training antibodies.

For the antigen component, none of the case-study antigens was an exact training-set match. In the Influenza/FNI17 case study, B/PK/2013, B/MA/2012, and A/DW/2021 showed relatively low protein-level similarities to the training antigens, 32.28%, 32.10%, and 21.60%, respectively, with query coverages of 93.08%, 99.23%, and 89.14%. A/SA/2016 and A/HK/2019 showed higher protein-level similarities, 77.09% and 91.45%, respectively, with near-complete query coverages of 99.54% and 99.48%, indicating that homologous influenza antigens were presented in the training set for these two cases. However, high overall sequence identity does not necessarily imply equivalent antibody-binding properties. Specifically, A/HK/2019 contains key substitutions (S245N and S247T) relative to 2AEQ that introduce an N245 glycosylation site. Previous studies have demonstrated that this specific glycosylation significantly reduces FNI17 binding (Momont et al., 2023). Therefore, despite high global sequence identity to training examples, such minimal but critical localized variations can drastically alter the binding behaviour. In contrast, the IL-33 antigen showed only 25.70% protein-level similarity to the training antigens at 91.82% query coverage.

In conclusion, although some influenza antigens had homologous training neighbours at the single-protein level, no case-study antigen was exactly present in the training set.

Table S14: **Complete antibody–antigen pair overlap and joint similarity against the training set.** × denotes no exact antibody–antigen pair overlap with the training set.

Case-study Complex	Exact Pair Overlap	Pair-level Joint Similarity	Number of Complexes
Influenza/FNI17			
FNI17–B/PK/2013	×	32.28%	1
FNI17–B/MA/2012	×	32.10%	1
FNI17–A/HK/2019	×	47.41%	1
FNI17–A/DW/2021	×	21.60%	1
FNI17–A/SA/2016	×	64.06%	1
IL-33/I7			
I7-WT and 19 variants–IL-33	×	25.70%	20

S10.3 Complete-complex leakage analysis

At the complete antibody–antigen pair level, no exact pair overlap was found for either case study. The maximum pair-level joint similarity was 64.06% for the five Influenza/FNI17 complexes and 25.70% for the 20 IL-33/I7 complexes, as summarized in Table S14. Compared with the individual-protein level results in Table S13, although A/HK/2019 had a highly similar antigen in the training set, with an individual-protein level similarity of 91.45%, the complete FNI17–A/HK/2019 complex had a pair-level joint similarity of only 47.41%. Similarly, A/SA/2016 had 77.09% individual-protein level similarity, whereas the corresponding FNI17–A/SA/2016 complex had a pair-level joint similarity of 64.06%.

In summary, these analyses show that none of the evaluated antibodies, antigens, or complete antibody–antigen pairs was exactly present in the training set. They also distinguish single-protein similarity from complete pair-level leakage: although two influenza antigens had homologous training neighbours, the corresponding FNI17–antigen complexes were absent from training and showed lower pair-level joint similarity than their antigen-only similarities. These findings indicate that the case-study results are unlikely to be attributable to exact sequence memorization or exact antibody–antigen pair overlap in the training data.

S11 Gradient-Magnitude Diagnostics for the Pretraining Objectives

To further assess whether any pretraining objective dominates the optimization, we performed the following additional analyses:

S11.1 Loss convergence and relative contributions

First, we recorded the values of L_{CE}, L_{INS}, and L_{Clus} throughout the 400 training epochs (Fig. S6A). After all three objectives became active at epoch 20, their mean loss values over epochs 20–399 were 2.07, 0.91, and 3.78, respectively. These corresponded to mean contributions of 30.63%, 13.49%, and 55.88% to the unweighted summed objective. Although L_{Clus} was the largest scalar component, it accounted for approximately 56.00% rather than nearly all of the objective, while L_{CE} and L_{INS} jointly contributed approximately 44.00%.

S11.2 Dynamic Weight Average (DWA) Strategy Effectiveness

Second, we incorporated the PU Pre-training stage of AbLWR (AbLWR_PU) with the DWA strategy, named AbLWR_PU_DWA, which utilizes DWA to dynamically assigns larger weights to the objectives exhibiting slower relative convergence. As shown in Fig. S6B, the DWA coefficients rapidly converged close to 1 : 1 : 1. Across epochs 20–399, the coefficients were 0.9999 ± 0.0019 (with the range of 0.9748–1.0084), 1.0001 ± 0.0040 (with the range of 0.9877–1.0695), and 1.0000 ± 0.0027 (with the range of 0.9557–1.0126) for L_{CE}, L_{INS}, and L_{Clus}, respectively. These results show that the dynamically learned coefficients remained close to equal weighting throughout the training process.

Furthermore, the performance of AbLWR_PU_DWA is found to achieve comparable performance as AbLWR_PU, as shown in Fig. S6C. Using validation AUC for checkpoint selection, AbLWR_PU achieved validation and test AUC values of 0.76 and 0.76, respectively, whereas the AbLWR_PU_DWA model achieved values of 0.76 and 0.77.

Subsequently, to evaluate whether DWA strategy used in the PU pre-training stage improves the downstream ranking task, we initialized the ranking model with the AbLWR_PU_DWA representation and fine-tuned it using the same procedure and data as the AbLWR. For convenience, we refer to this fine-tuned model as AbLWR_DWA. The resulting performance is summarized in Table S15.

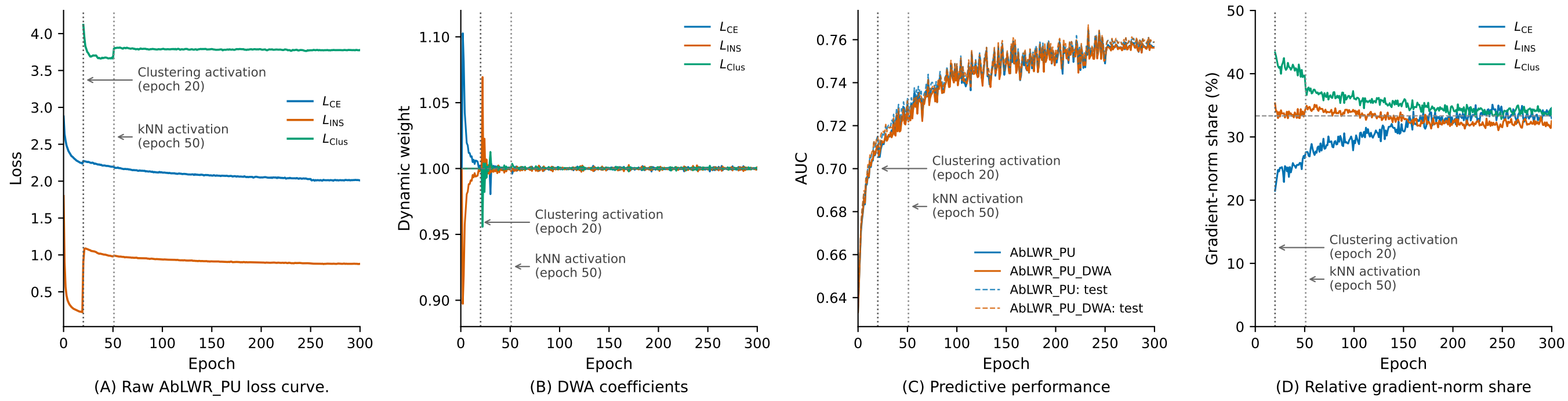

Figure S6: **Convergence, dynamic weighting, predictive performance, and gradient contributions of the pretraining objectives.** (**A**) L_{CE}, L_{INS}, and L_{Clus} values under equal weighting. (**B**) Dynamic Weight Average coefficients assigned to the three objectives. (**C**) Validation and test AUC of the AbLWR_PU and AbLWR_PU_DWA models. (**D**) Relative gradient-norm shares of the three objectives with respect to the shared antibody and antigen GCN encoders. The dashed horizontal line in panel D denotes the reference value of 33.30% expected under equal gradient-norm contributions.

Table S15: **Performance comparison between AbLWR and AbLWR_DWA.**

Model Variant	FRA (%)	K_τ	PRA (%)	PAU (%)	P@1 (%)
**AbLWR (Ours)**	**21.28**	**0.60**	**79.96**	**80.14**	**57.73**
AbLWR_DWA	21.05	0.59	79.43	79.61	57.39

Compared with the AbLWR, AbLWR_DWA yielded slightly lower performance across all evaluated metrics, including FRA (21.05% vs. 21.28%), K_τ (0.59 vs. 0.60), PRA (79.43% vs. 79.96%), PAU (79.61% vs. 80.14%), and P@1 (57.39% vs. 57.73%). The differences were small, with absolute changes of 0.23 percentage points or less for FRA, 0.01 for K_τ, and approximately 0.34–0.53 percentage points for the remaining ranking metrics. These results indicate that DWA strategy used in the pre-training stage does not provide a measurable downstream advantage over equal weighting in this experiment.

S11.3 Gradient norms on shared encoder

Finally, to directly examine gradient dominance, we measured the gradient norm produced by each objective with respect to the shared antibody and antigen GCN encoders. Gradient norms were sampled every ten mini-batches and aggregated by epoch. We then calculated the relative gradient-norm contribution of each objective, defined as its gradient norm divided by the sum of the gradient norms of the three objectives.

Over epochs 20–399, the median gradient norms were 2.85, 2.86, and 3.03 for L_{CE}, L_{INS}, and L_{Clus}, respectively. Their median relative gradient-norm contributions were 32.70%, 32.50%, and 34.50%, respectively (Fig. S6D). Thus, although the L_{Clus} objective had a slightly larger median gradient norm, the three objectives produced gradients of comparable magnitude, and no component consistently dominated the shared-encoder updates. All three gradient norms remained within the same order of magnitude throughout training.

Table S16: **Gradient-magnitude statistics for the three pretraining objectives over epochs 20–399.** The gradient-norm share is normalized by the sum of the three objective-specific gradient norms.

Objective	Median gradient norm	Median gradient-norm share
L_{CE}	2.85	32.70%
L_{INS}	2.86	32.51%
L_{Clus}	3.03	34.55%
Largest-to-smallest ratio	1.28	–

Together, the loss trajectories, gradient-magnitude analysis, and DWA sensitivity analysis demonstrate that equal weighting is a stable empirical choice in this experiment. The raw scalar loss values are not identical across objectives, but their effects on the shared encoder are comparable in magnitude.

References

C. J. Burges. From ranknet to lambdarank to lambdamart: An overview. *Learning*, 11(23-581):81, 2010.

M. Cox, T. P. Peacock, W. T. Harvey, J. Hughes, D. W. Wright, C.-. G. U. C.-U. Consortium, B. J. Willett, E. Thomson, R. K. Gupta, S. J. Peacock, et al. Sars-cov-2 variant evasion of monoclonal antibodies based on in vitro studies. *Nature Reviews Microbiology*, 21(2):112–124, 2023.

J. Dunbar, K. Krawczyk, J. Leem, T. Baker, A. Fuchs, G. Georges, J. Shi, and C. M. Deane. Sabdab: the structural antibody database. *Nucleic Acids Research*, 42(D1):D1140–D1146, 2014.

E. Engelhart, R. Emerson, L. Shing, C. Lennartz, D. Guion, M. Kelley, C. Lin, R. Lopez, D. Younger, and M. E. Walsh. A dataset comprised of binding interactions for 104,972 antibodies against a sars-cov-2 peptide. *Scientific Data*, 9(1): 653, 2022.

L. Fu, B. Niu, Z. Zhu, S. Wu, and W. Li. Cd-hit: accelerated for clustering the next-generation sequencing data. *Bioinformatics*, 28(23):3150–3152, 2012.

L. Goldstein, Y. Chen, J. Wu, S. Chaudhuri, Y. Hsiao, K. Schneider, K. Hoi, Z. Lin, S. Guerrero, B. Jaiswal, et al. Massively parallel singlecell b-cell receptor sequencing enables rapid discovery of diverse antigen-reactive antibodies. commun biol. 2019; 2: 304, 2019.

O. Gotoh. An improved algorithm for matching biological sequences. *Journal of molecular biology*, 162(3):705–708, 1982.

A. J. Greaney, T. N. Starr, and J. D. Bloom. An antibody-escape estimator for mutations to the sars-cov-2 receptor-binding domain. *Virus Evolution*, 8(1):veac021, 2022.

S. Henikoff and J. G. Henikoff. Amino acid substitution matrices from protein blocks. *Proceedings of the national academy of sciences*, 89(22):10915–10919, 1992.

Y. Hu, Z.-A. Huang, R. Liu, X. Xue, X. Sun, L. Song, and K. C. Tan. Source free semi-supervised transfer learning for diagnosis of mental disorders on fmri scans. *IEEE Transactions on Pattern Analysis and Machine Intelligence*, 45 (11):13778–13795, 2023. doi: 10.1109/TPAMI.2023.3298332.

J. Jankauskaitė, B. Jiménez-García, J. Dapkūnas, J. Fernández-Recio, and I. H. Moal. Skempi 2.0: an updated benchmark of changes in protein–protein binding energy, kinetics and thermodynamics upon mutation. *Bioinformatics*, 35(3): 462–469, 2019.

J. Lee, Y. Lee, J. Kim, A. Kosiorek, S. Choi, and Y. W. Teh. Set transformer: A framework for attention-based permutation-invariant neural networks. In *Proceedings of the International Conference on Machine Learning*, pages 3744–3753. PMLR, 2019.

Z. Lin, H. Akin, R. Rao, B. Hie, Z. Zhu, W. Lu, N. Smetanin, R. Verkuil, O. Kabeli, Y. Shmueli, et al. Evolutionary-scale prediction of atomic-level protein structure with a language model. *Science*, 379(6637):1123–1130, 2023.

C. Liu, A. Pelissier, Y. Shao, L. Denzler, A. C. Martin, B. Paige, and M. R. Martínez. Abrank: A benchmark dataset and metric-learning framework for antibody-antigen affinity ranking. *arXiv preprint arXiv:2506.17857*, 2025.

K. Michalewicz, M. Barahona, and B. Bravi. Antipasti: Interpretable prediction of antibody binding affinity exploiting normal modes and deep learning. *Structure*, 32(12):2422–2434, 2024.

C. Momont, H. V. Dang, F. Zatta, K. Hauser, C. Wang, J. di Iulio, A. Minola, N. Czudnochowski, A. De Marco, K. Branch, et al. A pan-influenza antibody inhibiting neuraminidase via receptor mimicry. *Nature*, 618(7965): 590–597, 2023.

S. B. Needleman and C. D. Wunsch. A general method applicable to the search for similarities in the amino acid sequence of two proteins. *Journal of molecular biology*, 48(3):443–453, 1970.

T. Nguyen, H. Le, T. P. Quinn, T. Nguyen, T. D. Le, and S. Venkatesh. Graphdta: predicting drug–target binding affinity with graph neural networks. *Bioinformatics*, 37(8):1140–1147, 2021.

Y. Park and E. M. Marcotte. Flaws in evaluation schemes for pair-input computational predictions. *Nature methods*, 9 (12):1134–1136, 2012.

S. Pittala and C. Bailey-Kellogg. Learning context-aware structural representations to predict antigen and antibody binding interfaces. *Bioinformatics*, 36(13):3996–4003, 2020.

P. Rawat, D. Sharma, R. Prabakaran, F. Ridha, M. Mohkhedkar, V. Janakiraman, and M. M. Gromiha. Ab-cov: a curated database for binding affinity and neutralization profiles of coronavirus-related antibodies. *Bioinformatics*, 38 (16):4051–4052, 2022.

J. A. Ruffolo, J. J. Gray, and J. Sulam. Deciphering antibody affinity maturation with language models and weakly supervised learning. *arXiv preprint arXiv:2112.07782*, 2021.

J. A. Ruffolo, L.-S. Chu, S. P. Mahajan, and J. J. Gray. Fast, accurate antibody structure prediction from deep learning on massive set of natural antibodies. *Nature Communications*, 14(1):2389, 2023.

J. Schymkowitz, J. Borg, F. Stricher, R. Nys, F. Rousseau, and L. Serrano. The foldx web server: an online force field. *Nucleic Acids Research*, 33(suppl_2):W382–W388, 2005.

A. Shanehsazzadeh, S. Bachas, M. McPartlon, G. Kasun, J. M. Sutton, A. K. Steiger, R. Shuai, C. Kohnert, G. Rakocevic, J. M. Gutierrez, et al. Unlocking de novo antibody design with generative artificial intelligence. *BioRxiv*, pages 2023–01, 2023.

T. F. Smith, M. S. Waterman, et al. Identification of common molecular subsequences. *Journal of molecular biology*, 147(1):195–197, 1981.

V. Ullanat, B. Jing, S. Sledzieski, and B. Berger. Learning the language of protein-protein interactions. *Nature Communications*, 2026.

R. Wang, F. Wu, J. Shi, Y. Song, Y. Kong, J. Ma, B. He, Q. Yan, T. Ying, P. Zhao, et al. A generative foundation model for antibody design. *BioRxiv*, pages 2025–09, 2025.

Y. Yan, H. Tao, J. He, and S.-Y. Huang. The hdock server for integrated protein–protein docking. *Nature Protocols*, 15 (5):1829–1852, 2020.

H. Yoon, J. Macke, A. P. West Jr, B. Foley, P. J. Bjorkman, B. Korber, and K. Yusim. Catnap: a tool to compile, analyze and tally neutralizing antibody panels. *Nucleic Acids Research*, 43(W1):W213–W219, 2015.
